# Primate tarsal bones from Egerkingen, Switzerland, attributable to the middle Eocene adapiform *Caenopithecus lemuroides*

**DOI:** 10.7717/peerj.1036

**Published:** 2015-06-23

**Authors:** Erik R. Seiffert, Loïc Costeur, Doug M. Boyer

**Affiliations:** 1 Department of Anatomical Sciences, Stony Brook University, Stony Brook, NY, USA; 2 Department of Geosciences, Naturhistorisches Museum Basel, Basel, Switzerland; 3 Department of Evolutionary Anthropology, Duke University, Durham, NC, USA

**Keywords:** Adapidae, *Afradapis*, Talus, Strepsirrhini, Calcaneus, Astragalus, Europe, Africa, Locomotion, Phylogeny

## Abstract

The middle Eocene species *Caenopithecus lemuroides*, known solely from the Egerkingen fissure fillings in Switzerland, was the first Paleogene fossil primate to be correctly identified as such (by Ludwig Rütimeyer in 1862), but has long been represented only by fragmentary mandibular and maxillary remains. More recent discoveries of adapiform fossils in other parts of the world have revealed *Caenopithecus* to be a biogeographic enigma, as it is potentially more closely related to Eocene adapiforms from Africa, Asia, and North America than it is to any known European forms. More anatomical evidence is needed, however, to provide robust tests of such phylogenetic hypotheses. Here we describe and analyze the first postcranial remains that can be attributed to *C. lemuroides*—an astragalus and three calcanei held in the collections of the Naturhistorisches Museum Basel that were likely recovered from Egerkingen over a century ago. Qualitative and multivariate morphometric analyses of these elements suggest that *C. lemuroides* was even more loris-like than European adapines such as *Adapis* and *Leptadapis*, and was not simply an adapine with an aberrant dentition. The astragalus of *Caenopithecus* is similar to that of younger *Afradapis* from the late Eocene of Egypt, and parsimony and Bayesian phylogenetic analyses that include the new tarsal data strongly support the placement of *Afradapis* and *Caenopithecus* as sister taxa to the exclusion of all other known adapiforms, thus implying that dispersal between Europe and Africa occurred during the middle Eocene. The new tarsal evidence, combined with previously known craniodental fossils, allows us to reconstruct *C. lemuroides* as having been an arboreal and highly folivorous 1.5–2.5 kg primate that likely moved slowly and deliberately with little or no capacity for acrobatic leaping, presumably maintaining consistent powerful grasps on branches in both above-branch and inverted postures.

## Introduction

*Caenopithecus* is a phylogenetically and biogeographically enigmatic adapiform primate whose fossil record is restricted to middle Eocene (Lutetian, ∼43 Ma, MP13b) fissure fillings in the commune of Egerkingen, Canton Solothurn, northern Switzerland (Godinot, 1998; Rütimeyer, 1862; Stehlin, 1916). Upper molars of the type and only species, *Caenopithecus lemuroides*, were described by Rütimeyer (1862), and later Stehlin (1916) described parts of the lower dentition, mandible, additional parts of the upper dentition, and the orbital region. These limited remains show that *C. lemuroides* was a relatively large adapiform—having second lower molars that are about the same length as those of the extant lemurids *Eulemur fulvus* and *Prolemur simus* (Kay et al., 2004)—and had a fused mandibular symphysis, large canine teeth, very small P^2^/_2_, simple P^3−4^/_3−4_, quadrate upper molars with distinct hypocones and mesostyles, and narrow lower molars with elongate crests and well-developed metastylids. The maxillary remains of *C. lemuroides* show that the species had a postorbital bar, but no postorbital closure. Analysis of lower molar shearing crests suggests that *C. lemuroides* was a dedicated folivore (Kay et al., 2004).

Other remains of *Caenopithecus* have been reported since the publication of Stehlin (1916), but are not universally accepted as belonging to that genus—Franzen (1994) identified partial skeletons from the Geiseltal and Messel deposits as belonging to a new species of *Caenopithecus* (i.e., *Caenopithecus* “*neglectus*”), but these are now the type specimens of *Godinotia neglecta* (Franzen, 2000) and *Darwinius masillae* (Franzen et al., 2009), and Godinot (1988) has expressed doubt about the presence of *Caenopithecus* at Bouxwiller, France, that was reported by Jaeger (1971). Stehlin also attributed an astragalus from Egerkingen (NMB Eh 741) to *C. lemuroides*, but this element is probably that of a non-primate hyaenodontidan ‘creodont’ (see also Decker & Szalay, 1974).

In an unpublished doctoral dissertation, Dagosto (1986) attributed an astragalus and three calcanei from the original Egerkingen collections held at the Naturhistorisches Museum Basel (NMB) to *C. lemuroides* and briefly described those elements.^1^1We were unaware of Dr. Dagosto’s earlier work at the time that this manuscript was submitted for review, and we thank Dr. DL Gebo for bringing this to our attention. Here we expand on Dagosto’s earlier work by (1) providing quantitative justification for attribution of these tarsals to *C. lemuroides*, (2) describing and comparing the Egerkingen tarsals in greater detail, notably making comparisons with several specimens that have been discovered since Dagosto’s work was completed in 1986; (3) analyzing the more complete specimens using multivariate and other morphometric techniques; and (4) including *C. lemuroides* in phylogenetic analyses alongside numerous other living and extinct primates. The tarsals of *C. lemuroides* provide important new insights into the locomotor adaptations and relationships of this mysterious primate.

### History of study

Rütimeyer (1862) identified *Caenopithecus* as a primate (and in so doing was the first to correctly identify a fossil primate of Paleogene age (Stehlin, 1916)); with little comparative material available for study, he (and later Forsyth Major, 1872) was impressed by upper molar features that *Caenopithecus* shared with extant howler monkeys (*Alouatta*). The identification of *Caenopithecus* as a primate was subsequently questioned by various authorities (e.g., Delfortrie, 1873), but Gervais (1873) considered *Caenopithecus* to be a synonym of *Adapis* (see also Flower, 1876; Forbes, 1894; Schlosser, 1887), and Gaudry (1878) argued that *Caenopithecus* was aligned with lemurs, drawing attention to dental features that the genus shared with extant *Hapalemur* and *Lemur*. Stehlin (1916) noted additional features of the lower dentition and mandible that *Caenopithecus* shared with the few additional European adapiforms that were known by the time of his study. With the benefit of an expanding North American fossil record, Stehlin also pointed out dental similarities to *Shoshonius* and *Washakius*, which are now considered to be distantly related washakiin omomyiforms. Abel (1931) and Le Gros Clark (1959) believed *Caenopithecus* to be a tarsioid,^2^2It should be noted that Abel’s “Tarsioidea” also included taxa that are now considered to be plesiadapiforms (e.g., *Paromomys*, *Carpolestes*) or adapiforms (*Anchomomys*, *Periconodon*, *Pronycticebus*). Other adapiforms (*Adapis*, *Pelycodus*, *Notharctus*, *Protoadapis*) were included in his Lemuroidea. and Gregory (1920) later suggested that the genus might be related to indrioid lemurs, but these are minority views; most debates subsequent to the publications of Stehlin (1912) and Stehlin (1916) have focused on whether *Caenopithecus* is more closely related to adapines such as *Adapis* and *Leptadapis* (e.g., Godinot, 1998; Rose, Godinot & Bown, 1994; Russell, Louis & Savage, 1967; Simons, 1972; Simpson, 1940; Szalay & Delson, 1979) or to some “cercamoniine” adapiform such as *Europolemur* or *Protoadapis* (Franzen, 1994; Gingerich, 1977; Remane, 1956; Simons, 1962; Weigelt, 1933). In her unpublished doctoral dissertation, Dagosto (1986) briefly described and figured the tarsals analyzed here, attributed them to *C. lemuroides*, and concluded that they “support the hypothesis of a close relationship between *Adapis*, *Leptadapis*, and *Caenopithecus*” (p. 198) and that “*Adapis*, *Leptadapis*, and *Caenopithecus*…have abandoned the primitive euprimate emphasis on leaping and have evolved locomotor modes which stress quadrupedal locomotion and climbing” (pp. 201–202).

The study of *Caenopithecus* has been further complicated in recent years by the discovery of several geographically far-flung taxa, such as *Aframonius*, in the late Eocene of Africa (Simons, Rasmussen & Gingerich, 1995), and the poorly known *Adapoides*, in the middle Eocene of Asia (Beard et al., 1994), that have been identified as close relatives of *Caenopithecus* and placed in Caenopithecinae (a subfamily of the otherwise strictly European Adapidae; Godinot (1998)). In proposing this group, Godinot (1998) also included the problematic middle Eocene genus *Mahgarita* from North America. Membership has since expanded to include late Eocene *Afradapis* from Egypt (Boyer, Seiffert & Simons, 2010; Seiffert et al., 2009), and possibly *Mescalerolemur* (from the middle Eocene of Texas; Kirk & Williams, 2011) and *Darwinius* (from the middle Eocene of Europe; Franzen et al., 2009; see phylogenetic analysis of Seiffert et al., 2009). *Mahgarita* and *Mescalerolemur* are the only non-notharctine adapiforms known from North America, and, like *Caenopithecus*, their geographic origins remain mysterious (Kirk & Williams, 2011). Fleagle (2013) placed all of these taxa, including middle Eocene European *Europolemur* and *Godinotia*, into a new family, Caenopithecidae. Here we use the nomen Caenopithecinae and use it to refer to *Caenopithecus* and its demonstrable near relatives (*Afradapis* and *Aframonius*), because the phylogenetic analyses that we present here call into question the monophyly of an assemblage that includes these taxa as well as *Mahgarita* and *Mescalerolemur* (see also Kirk & Williams, 2011). We refer to the clade that includes *Adapis* and *Leptadapis* (the monophyly of which is consistently supported by our phylogenetic analyses) as Adapinae.

The higher-level affinities of caenopithecines and *Mahgarita* continues to be debated; authorities have identified some of these species as stem anthropoids or stem haplorhines (Franzen et al., 2009; Rasmussen, 1990; Rasmussen, 1994; Simons, Rasmussen & Gingerich, 1995) while others have considered some or all of these species to be most parsimoniously interpreted as stem strepsirrhines (Dagosto & Gebo, 1994; Godinot, 1998; Kay, Ross & Williams, 1997; Kirk & Williams, 2011; Maiolino et al., 2012; Marivaux et al., 2013; Ni et al., 2013; Rose et al., 2009; Ross, Williams & Kay, 1998; Seiffert et al., 2009; Seiffert et al., 2005). Until recently, the only evidence that could be brought to bear on this debate was the dental anatomy of these taxa, and the poorly preserved crania of *Mahgarita* (see for instance the different interpretations of Rasmussen, 1990, and Ross, 1994), but the recent discovery of an astragalus of *Afradapis* (Boyer, Seiffert & Simons, 2010) showed that taxon to be remarkably strepsirrhine-like in its tarsal morphology, contrasting strongly with that which would be expected along the haplorhine or anthropoid stem lineages (see for instance Boyer & Seiffert, 2013). The astragalus of *Afradapis* shows several similarities to the astragalus from the Egerkingen collections that we describe here, bolstering the evidence for its attribution to *Caenopithecus*, which has been placed as the sister taxon of *Afradapis* in multiple phylogenetic analyses (Boyer & Seiffert, 2013; Boyer, Seiffert & Simons, 2010; Kirk & Williams, 2011; Ni et al., 2013; Seiffert et al., 2009). We further test all of these phylogenetic hypotheses by adding the character data from the Egerkingen tarsals to the morphological character matrix that has most recently been employed by Boyer et al. (2015b), which we analyze using both parsimony and Bayesian approaches.

## Materials and Methods

### Attribution

The tarsals described here derive from the Egerkingen fissure fills, but provenance is only documented for one specimen, NMB Eh 719, which was recovered from the *γ* (= “Gamma”) fissure. In her dissertation, Dagosto (1986) stated that all of the specimens are from the *γ* fissure, suggesting that locality information for the specimens was available in 1986 but has since been lost. On the basis of articular compatibility of the astragalus (NMB En.270) with the calcaneus NMB Eh 719, as well as the similar size, color, preservation, and peculiar morphology of all three calcanei (i.e., NMB Eh 719, En.268, and En.269), we consider it highly probable that all of the tarsals described here belong to the same species, and so are likely to derive from the same fissure that Eh 719 was recovered from (i.e., fissure *γ*, where the two relatively large adapiforms *C. lemuroides* and *Leptadapis priscus* occur; note that Dagosto (1986), was unaware that remains of *L. priscus* have also been recovered from fissure *γ*, and so thought that the much smaller species *Microadapis sciureus* was the only other option for attribution). We employ measures of relative abundance (by comparing the number of craniodental specimens attributed to each large adapiform species known from Egerkingen *γ*) and regressions of dental and tarsal variables (i.e., astragalar trochlear width and calcaneo-cuboid joint size to M_2_ size across a sample of living primates) to determine the most probable species attribution. Original scans and digital models of all specimens are available on MorphoSource (www.morphosource.org); a DOI for each specimen is provided in Table 1.

**Table 1:** DOIs and MorphoSource ID numbers for scans and digital models of the Egerkingen tarsals.

DOI	Morphosource ID	File name	File type	Specimen number	Element
10.17602/M2/M5397	M5963-5397	NMB-En-270_M5963-5397.zip	Zipped tiff stack	NMB En.270	Left astragalus
10.17602/M2/M5398	M5963-5398	NMB-En-270_M5963-5398.ply	Smoothed mesh file	NMB En.270	Left astragalus
10.17602/M2/M5399	M5964-5399	NMB-Eh-719_M5964-5399.zip	Zipped tiff stack	NMB Eh 719	Right calcaneus
10.17602/M2/M5400	M5964-5400	NMB-Eh-719_M5964-5400.ply	Smoothed mesh file	NMB Eh 719	Right calcaneus
10.17602/M2/M5401	M5965-5401	NMB-En-268_M5965-5401.zip	Zipped tiff stack	NMB En.268	Right calcaneus
10.17602/M2/M5402	M5965-5402	NMB-En-268_M5965-5402.ply	Smoothed mesh file	NMB En.268	Right calcaneus
10.17602/M2/M5403	M5966-5403	NMB-En-269_M5966-5403.zip	Zipped tiff stack	NMB En.269	Left calcaneus
10.17602/M2/M5404	M5966-5404	NMB-En-269_M5966-5404.ply	Smoothed mesh file	NMB En.269	Left calcaneus

We gathered comparative data on tarsal facet size and M_2_ size from extant and fossil primates (Appendix S1 and S2) in order to determine whether the Egerkingen tarsal bones predict tooth sizes more similar to those of *C. lemuroides* or *L. priscus*. To this end, we regressed natural log-transformed lower second molar (M_2_) area (= maximum mesiodistal length multiplied by maximum buccolingual breadth) on natural log-transformed astragalar trochlear width (shown to be highly correlated with body mass in Dagosto & Terranova (1992: their measure “A4”)) and calcaneo-cuboid facet area (= cuboid facet length times cuboid facet width, another measure shown to be highly correlated with body mass by Dagosto & Terranova (1992: their “Index 6”) and the best calcaneal predictor of body mass found by Yapuncich, Gladman & Boyer (2015)). Though M_2_ area may not be as robust a body mass proxy as M_1_ area (Gingerich, Smith & Rosenberg, 1982), it is a measure that is known in *C. lemuroides* and *L. priscus*, and is arguably more removed from functional/evolutionary pressures on tooth size and morphology that affect the adjacent molars. We also estimate the body mass of the Egerkingen primate using the equations derived from regressions of tarsal facet areas on body mass that were recently published by Yapuncich, Gladman & Boyer (2015).

Our null hypothesis for these analyses was that the Egerkingen tarsals belong to *C. lemuroides*, on the basis of the expression of morphology that would be consistent with that species’ demonstrated phylogenetic proximity to *A. longicristatus*, but not clearly consistent with attribution to a primitive adapine such as *L. priscus*, whose close relatives (including the alleged congener of *L. priscus*, *Leptadapis magnus*) differ in clear and quantifiable ways. If true, the tarsal dimensions (i.e., those of the astragalus NMB En.270 and the best-preserved calcaneus from Egerkingen, NMB Eh 719) should predict an M_2_ size matching that of teeth identified as belonging to *C. lemuroides*. Our extant sample includes 33 species means, in which all individuals used in computing those means included molar, astragalar, and calcaneal measurements (except for *Galagoides demidoff*, for which the molar data came from different specimens than those that provided the tarsal data). The sample also includes 30 fossil taxa, of which nine specimens have associated molar and tarsal data (Appendix S2). We also computed 95% prediction intervals as the limiting envelope within which the actual molar area must fall to be considered plausibly attributable to the postcranial specimen generating the estimate. Regressions were run in PAST.exe (Hammer, Harper & Ryan, 2001). 95% confidence limits on the prediction interval of tooth size from postcranial element dimensions were generated (using equation 17.29 of Zar, 1984). Note that prediction intervals that take into account the Prediction Standard Error (PSE) of the data (or the ‘scatter’) around the regression line are more appropriate than a confidence interval based on standard error in the regression parameters (e.g., Chatterjee & Simonoff, 2013). Prediction intervals intuitively must be broad enough to incorporate most of the data points used to construct the regression, whereas confidence intervals based on error in regression parameters typically exclude many more data points used to create the regression, indicating that these limits are not appropriate indicators of whether a given set of (for instance) postcranial and molar dimensions are closely associated.

**Figure 1:**
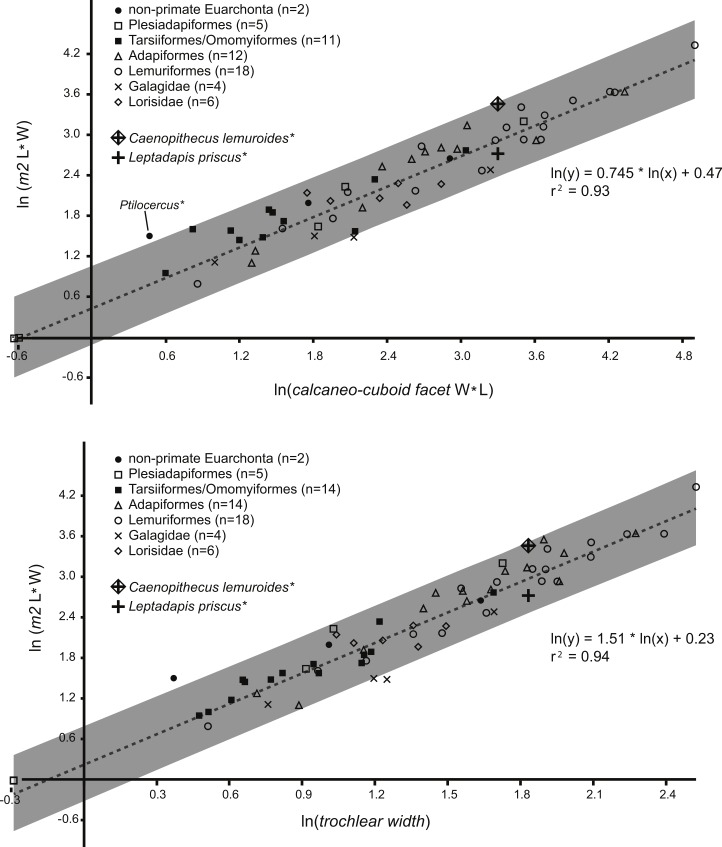
Regression of tooth size on tarsal facet size. Above, regression of the natural log of lower second molar area (length times width) on the natural log of calcaneo-cuboid facet area (length times width), with data from 51 living and extinct primates and seven non-primate euarchontans. *Solid circles*, extant non-primate euarchontans; *open squares*, plesiadapiforms; *solid squares*, tarsiers and extinct omomyiforms; *open triangles*, extinct adapiforms; *open circles*, extant lemuriforms; “x,” extant galagids; *open diamonds*, extant lorises; “+,” natural log of calcaneo-cuboid facet area of NMB Eh 719 plotted relative to the natural log of second lower molar size of *Leptadapis priscus*; “+” *enclosed in a diamond*, natural log of the calcaneo-cuboid facet area of NMB Eh 719 plotted relative to the natural log of second lower molar size of *Caenopithecus lemuroides*. See figure for regression equation and *r*^2^. Below, regression of the natural log of lower second molar area (length times width) on the natural log of astragalar trochlear width, with data from 63 living and extinct primates and seven non-primate euarchontans. Symbols as above; “+,” natural log of the trochlear width of NMB En.270 plotted relative to the natural log of second lower molar size of *Leptadapis priscus*; “+” *enclosed in a diamond*, natural log of trochlear width of NMB En.270 plotted relative to the natural log of second lower molar size of *Caenopithecus lemuroides*. See figure for regression equation and *r*^2^. Note that the extant pen-tailed tree shrew *Ptilocercus* shows a tooth-tarsal scaling relationship that differs from that of living and extinct primates; for this reason it was excluded from the regression equation.

In plotting adapiform M_2_ area and tarsal measurements among those of other taxa (Fig. 1), we observed that larger species (i.e., those at or above “Kay’s Threshold” of 500 g) tend to have higher residuals. Therefore, instead of expecting the “owner” of NMB En.270 and NMB Eh 719 to have a small residual between its actual value and predicted value, we reasoned that it would more likely to exhibit a residual value close to the average residual value shown by other large adapiforms in the sample. To compare residuals among adapiforms, we first ran a regression that excluded large adapiforms (i.e., ten taxa were excluded from the trochlear width regression, and eight were excluded from the calcaneo-cuboid facet regression). We re-computed adapiform residuals relative to this new line and then compared the values of the M_2_ area residuals generated by the tarsal specimens under investigation to these populations using one sample *t*-tests, where the residual values of the tarsal dimensions were treated as the test values.

Finally, to address the possibility that the astragalus NMB En.270 and the calcaneus NMB Eh 719 could have come from two different species rather than a single species, we noted that for dentally-associated elements, molar residuals from the calcaneus and astragalus were strongly correlated. To quantify this correlation we converted residuals from the original regressions into *z*-scores. For each taxon occurring in both regressions (*n* = 57), we computed the absolute value of the differences between residuals generated from the trochlear width and calcaneo-cuboid joint area regressions. We found these differences to be small in general, and hypothesized that if both tarsals came from a single species, the difference in M_2_ residuals should be within the 95% confidence interval of the differences exhibited by the extant sample. If the tarsals came from two different species, there is no way to constrain how much difference should exist between the two sets of residuals when the bones are treated as if they came from a single species. Thus this test can potentially refute the hypothesis that both bones came from a single species (if the difference is outside the observed range), but not that they came from two species.

### Multivariate morphometric analysis of primate astragali

In order to quantitatively assess the phenetic affinities of the Egerkingen tarsals, we undertook a principal components analysis of a set of linear and angular measurements taken on digital models of 25 primate and 27 non-primate astragali. The dataset was first developed by Boyer (2009), and later augmented by Boyer, Seiffert & Simons (2010) and Chester et al. (2015). The astragalus described here (NMB En.270) lacks most of the head, and is abraded in such a way that not all measurements in the dataset could be taken. From an original set of 18 linear measurements, we were able to take 11 (specifically 2, 4–8, 10–14 from Boyer, Seiffert & Simons, 2010, some of which (4, “Fibular facet maximum dorsoplantar height”; 5, “Fibular facet proximodistal length”; 7, “Lateral tibial facet maximum mediolateral width”; and 12, “Flexor fibularis groove mediolateral width”) were estimated due to abrasion along the lateral margin of the lateral tibial facet (for 4, 5, and 7), and along the plantar surface of the medial tubercle buttressing the groove for flexor fibularis (for 12). Of six angular measurements, we were able to take three (20–22) (Appendix S3). All linear measurements were converted to shape ratios by dividing each measurement by a geometric mean (based on 10 measurements—4–8, 10–13, 15) and then log transforming those ratios. Angular measurements were converted to radians. A principal components analysis of these data was undertaken using the program PAST. See Appendix S3 for the complete dataset.

### Quantification of flexor fibularis groove depth

We used digital models of the astragali of 52 crown strepsirrhine individuals (20 genera, including three subfossil lemuriform genera) and seven adapiform genera to quantify the depth of the flexor fibularis groove along the most anterior extent of its plantar exposure. Astragali were oriented with the plantar surface facing upward and in posterior view, such that the point marking the base of the trough of the flexor fibularis groove was aligned with the point marking the plantar apex of the navicular facet or sustentacular facet (whichever was visible in that view). This cross-sectional view of the flexor fibularis groove was therefore oriented roughly perpendicular to the anteroposteriorly oriented line of action of the flexor fibularis tendon along the plantar surface of the astragalus. In this view, the two peaks formed by (1) the medial tubercle buttressing the groove and (2) the most plantar projection of the anteromedial aspect of the ectal facet provided landmarks for two measurements that were taken simultaneously using the “Measure” tool in the program Geomagic—the linear width of the flexor fibularis groove, and (by clicking “Projection” in Geomagic) the length of the contour between the two points that were used to calculate linear width (which, in Geomagic, is not dependent on orientation but rather is the shortest distance between those two points along the contour). Surfaces with rendering artifacts, such as artificially roughened or “spikey” areas, were smoothed in Geomagic to ensure accuracy of the contour measurement. We used a simple ratio of the contour measurement to the linear width measurement to describe the depth of the groove; species with a ratio of 1 show no concavity of the groove, while increasing positive departures from that value reflect increasing concavity (and therefore tall walls constraining the passage of the tendon). In species with very little concavity of the groove, the two landmarks could be difficult to place (because there were no obvious “peaks”), but this is not of great concern to us because wherever the two points were placed in such species, the ultimate values for the ratio approached equality; our interest in taking this measurement was to detect marked departures from equality, and to identify taxa that had markedly concave flexor fibularis grooves along the plantar surface of the astragalus.

### Peroneal tubercle position in early fossil primates

We took three measurements along the proximodistal long axis of the calcaneus in order to quantify peroneal tubercle position and size across a sample of early fossil primates (1: length of the proximal segment; 2: distance from the proximal-most aspect of the calcaneal tuber to the distal-most projection of the peroneal tubercle; and 3: distance from the proximal-most aspect of the calcaneal tuber to the midpoint of the peroneal tubercle). The sample includes a total of 100 individuals, composed of 51 adapiform specimens (*Adapis* (*n* = 6), *Asiadapis* (*n* = 2), *Caenopithecus* (*n* = 3), *Cantius* (*n* = 16), *Leptadapis* (*n* = 8), *Marcgodinotius* (*n* = 5), *Notharctus* (*n* = 7), *Smilodectes* (*n* = 4)), 21 omomyiform specimens (*Arapahovius* (*n* = 3), *Hemiacodon* (*n* = 1), *Omomys* (*n* = 6), *Ourayia* (*n* = 1), *Shoshonius* (*n* = 1), *Teilhardina* (*belgica*, *n* = 8), *Tetonius* (*n* = 1)), nine stem anthropoid specimens (*Eosimias* (*n* = 6), Parapithecidae (*n* = 5), *Proteopithecus* (*n* = 1)), six plesiadapiform specimens (*Carpolestes* (*n* = 1), *Ignacius* (*n* = 1), *Nannodectes* (*n* = 1), *Plesiadapis* (*n* = 3)), three dermopteran specimens (*Cynocephalus* (*n* = 2), *Galeopterus* (*n* = 1)), and ten scandentian specimens (*Ptilocercus* (*n* = 3), *Tupaia* (*n* = 7)).

### Automated geometric analysis of primate calcanei

In order to compare overall shape of the best-preserved calcaneus from Egerkingen (NMB Eh 719) with that of other living and extinct primates, we used an automated morphometric procedure that requires no researcher supervision (i.e., no measurements, landmarks or anatomical axes need be supplied for bones included in the comparison) (Boyer et al., 2015a). We chose to take this approach in order to minimize the degree to which characterizations of shape affinities are dependent on measurements selected, or researcher observer error or bias. We would opt for this approach with the astragalus as well, but the method cannot be easily implemented for analysis of fragmentary bones at this time. In order to create 3D digital models of calcanei, 159 specimens representing 46 primate genera and 6 non-primate euarchontan genera were Micro-CT or laser scanned and processed in Avizo and Geomagic to create shell-like (i.e., without internal structure) mesh files representing only the external surface of each bone. All surface files are published on www.morphosource.org and can be directly downloaded, though the cleaned, shell-like versions are not necessarily represented (but are available on request). These 3D digital models were then analyzed using the fully automated 3D geometric morphometric algorithm *auto3dgm* (Boyer et al., 2015a), a MATLAB application (available on GitHub). The algorithm is also available as an R-package, which can currently be downloaded at the following URL with documentation and tutorials (www.stat.duke.edu/~sayan/auto3dgm/index.shtml). Components of the method are detailed in Boyer et al. (2015a); here we present a brief explanation of the protocol. The analysis down-samples each surface to a uniform number of evenly spread landmarks—in this case 256 points, which it then uses to find pairwise alignments via the Closest Iterative Points algorithm (Besl & McKay, 1992). We reduce the risk of incorrect alignments by specifying eight initial alignments that represent all combinations of the first three principal axes of variation in the landmark points. 1,200 points were used to represent each bone’s surface. The initial set of pairwise distances between bones of the sample is used to define a minimum spanning tree linking all bones. Point correspondences are propagated through this network, allowing proper alignment of disparate shapes. This propagation process results in a final landmark dataset, and revised pairwise distance measures between all surfaces. We then used the Procrustes distance matrix in a Multidimensional Scaling Analysis with the MATLAB function ‘mds.m’ to condense the variation into two dimensions (the landmark output could also have been analyzed in *morphologika*^2.5^).

### Phylogenetic analysis

We undertook multiple phylogenetic analyses to determine how the new character data from the Egerkingen tarsals influenced previous placements of *Caenopithecus* and other adapiforms. The phylogenetic analyses presented here build on a morphological character matrix that is based largely on the original work of Kay, Ross & Williams (1997), Ross, Williams & Kay (1998), Seiffert, Simons & Attia (2003) and Seiffert, Simons & Simons (2004), and which has been successively augmented by Seiffert et al. (2009), Seiffert et al. (2010) and Seiffert et al. (2005), Boyer, Seiffert & Simons (2010), Patel et al. (2012), Boyer & Seiffert (2013), Gladman et al. (2013), and, most recently, Boyer et al. (2015b). The matrix (Dataset S1) now includes 391 characters, and, with the addition of *Mescalerolemur*, a possible caenopithecine from the middle Eocene of Texas (Kirk & Williams, 2011), a total of 109 taxa. We undertook both parsimony and Bayesian analyses of this character matrix.

Two initial parsimony analyses were carried out using PAUP 4.10b10 (Swofford, 1998). For both, heuristic searches were run for 10,000 replicates with random addition sequence and the tree bisection and reconnection algorithm. For one of the two parsimony analyses, 256 characters whose states could be reasonably arranged into ordered (additive) morphoclines were treated as such. A subset of these ordered characters (209 total) had polymorphisms that were scored as intermediate states rather than scored using standard polymorphic scoring (i.e., (01)). These 209 characters were scaled so that transitions between “fixed” states were equal to a single step. In addition, we employed a molecular scaffold that constrained extant taxa to fit with the prevailing primate phylogeny based on molecular sequence data (specifically, the results of Springer et al. (2012)), and we constrained characters encoding premolar loss so that teeth that had previously been lost could not be regained. Another parsimony analysis was run with the molecular scaffold enforced, but with no assumptions about character ordering or premolar re-evolution—i.e., all characters were treated as unordered, with all transitions between states equal to a single step. Equally parsimonious trees recovered by these analyses are summarized here as strict consensus trees, and bootstrap support is provided, based on 1,000 pseudoreplicates (also calculated in PAUP).

Two Bayesian analyses were carried out using MrBayes 3.2.2 (Ronquist et al., 2012) and that program’s Mk model for morphological data. Both analyses were run on the CIPRES server (Miller, Pfeiffer & Schwartz, 2010). The same molecular scaffold as that used in the parsimony analyses was enforced in MrBayes using partial constraints. Analyses were run for 50 million generations, with four chains (three heated, one cold), sampling every 1,000 generations. Trees were summarized as a “halfcompat” consensus (50% majority-rule consensus) with a relative burn-in (25% of the samples). One analysis was run with all characters treated as unordered. While it would be ideal to run a Bayesian analysis of the matrix with all 256 characters treated as ordered as in the parsimony analysis described above, unfortunately MrBayes only allows multistate characters to be treated as ordered if they have six or fewer states, and 28 of the ordered characters in the parsimony analysis have >6 states. In order to run comparable parsimony and Bayesian analyses with all 256 characters treated as ordered, we removed the intermediate polymorphic states in the matrix and used standard polymorphic scoring. We consider this solution to be far from ideal, because it effectively renders those polymorphisms uninformative for phylogenetic reconstruction, but it is the only clear option that we could find for running comparable analyses while maintaining what we consider to be appropriate character state delimitations (the alternative being to collapse adjacent states into the same state, which would also lead to loss of information). In all of the Bayesian analyses coding was set to “variable” (lset coding = variable), which led to the exclusion of invariant or parsimony uninformative characters. The parsimony analysis of this modified matrix was run in the same way as the other parsimony analyses, as described above. We also run all of the same analyses, with all of the same assumptions, with the Egerkingen tarsals scored as belonging to *Leptadapis priscus*, the other large adapiform at Egerkingen fissure *γ*, in order to determine whether attribution to this taxon (rather than to *Caenopithecus*) has an impact on phylogenetic relationships among adapiforms.

### Micro-CT scanning

The Egerkingen tarsals described here were micro-CT scanned at the American Museum of Natural History’s Microscopy and Imaging Facility, using a Phoenix brand v/tome/x s240 micro-CT scanner. High resolution scan and photographic imagery utilized here are available through MorphoSource.org. Scans of taxa used in comparative analyses and details on scanning facility, scanning resolution and energy settings are also largely available through MorphoSource.org. Additional details are available in appendix tables or supplementary information of Boyer et al. (2013) and Boyer & Seiffert (2013).

## Results

### Attribution

On the basis of astragalar trochlear width and calcaneo-cuboid joint surface size relative to M_2_ size, the possibility of the Egerkingen primate tarsals being attributable to either *Anchomomys* cf. *pygmaeus* or *Necrolemur* cf. *zitteli* (both of which have been recovered from Egerkingen *γ*) can be confidently excluded. Of the remaining possibilities (*Caenopithecus lemuroides* or the dentally smaller *Leptadapis priscus*) Stehlin (1916) lists 22 specimens in the Egerkingen *γ* collection of *C. lemuroides* (an additional six are known from the “Huppersand”), but only two specimens of *L. priscus*. Using a more conservative metric, the minimum number of *C. lemuroides* individuals represented in the Egerkingen *γ* collection is six, while the minimum number of *L. priscus* individuals represented in Egerkingen *γ* collection is two. On the basis of abundance, the most likely attribution is to *C. lemuroides*. This is particularly true when the number of isolated tarsals is taken in account—i.e., it is much more likely that four isolated tarsals (all likely attributable to a single species) would be derived from the species represented by 22 non-tarsal specimens (*C. lemuroides*), than to the species represented by only two non-tarsal specimens (*L. priscus*).

Regressions of M_2_ area against tarsal dimensions showed high coefficients of determination of 0.94 (trochlear width) and 0.93 (calcaneo-cuboid facet size) (Fig. 1). The M_2_ area of *C. lemuroides* is within the computed prediction intervals for M_2_ area generated by both tarsal elements, though it has a high positive residual indicating that, if the tarsals are attributable to *C. lemuroides*, that species would have relatively small tarsal facets compared to M_2_ area (Fig. 1). The M_2_ area of *L. priscus* also falls within this interval, but with a fairly small and slightly negative residual. On this basis, the tarsals could belong to either species and would apparently be more likely candidates for attribution to *L. priscus*.

**Figure 2:**
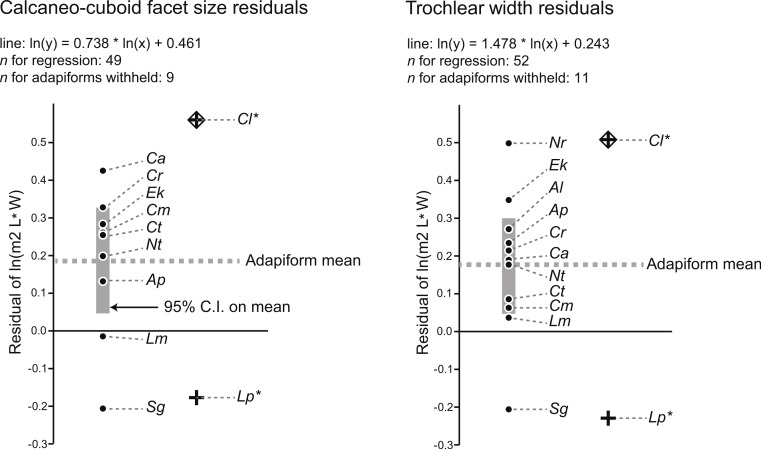
Residuals for the natural log of M_2_ area of adapiforms following recalculation of regression of the natural log of M_2_ area on tarsal facet area *excluding* those adapiform species. Note that *Caenopithecus lemuroides* (“Cl”) and *Leptadapis priscus* (“Lp”) have predicted values for the natural log of lower second molar area that fall outside of the 95% confidence interval on the mean for both calcaneocuboid facet area (above) and trochlear width (below). Other abbreviations: “Ap,” *Adapis parisiensis*; “Al,” *Afradapis longicristatus*; “Ca,” *Cantius abditus*; “Cm,” *Cantius mckennai*; “Cr,” *Cantius ralstoni*; “Ct,” *Cantius trigonodus*; “Ek,” *Europolemur klatti*; “Nr,” *Notharctus robustior*; “Nt,” *Notharctus tenebrosus*; “Lm,” *Leptadapis magnus*; “Sg,” *Smilodectes gracilis*. See figure for regression equations.

Using a modified regression that excludes large adapiforms, we then compared typical residual values of these adapiforms to those of the focal fossils. We found that the residual values of the two candidate owners of the Egerkingen tarsals are *both* well outside of the 95% confidence limits on the means of the residual value distributions exhibited by other large adapiforms (Fig. 2). Computing the significance of the difference between the focal fossil residual values and the means for the known associations, we find that, for the astragalus, the probability of attribution to *L. priscus* is lower (*t*-test of null hypothesis that the adapiform mean is equal to that of *L. priscus* residual of −0.23; adapiform mean is 0.18, 95% C.I. is 0.05–0.30; *t*-value = 7.30; *p* (null correct) = 0.00003) than to *C. lemuroides* (residual of 0.51; *t*-value = −5.94; *p* (null correct) = 0.0001). For the calcaneus, the probability of attribution to *C. lemuroides* is slightly lower (*t*-test of null hypothesis that adapiform mean is equal to the *C. lemuroides* residual of 0.57; adapiform mean is 0.19, 95% C.I. is 0.04–0.33; *t*-value = −5.85; *p* (null correct) = 0.0004) than attribution to *L. priscus* (residual of −0.18; *t*-value 5.67; *p* (null correct) = 0.0005). However, the differences are minimal in both cases, and again we note that NMB Eh 719 and NMB En.270 have strange (but morphologically and metrically compatible) articular surfaces, arguing against attribution to two different species of different dental size. Nonetheless, these results may lead one to question whether the bones belong to two different taxa.

**Figure 3:**
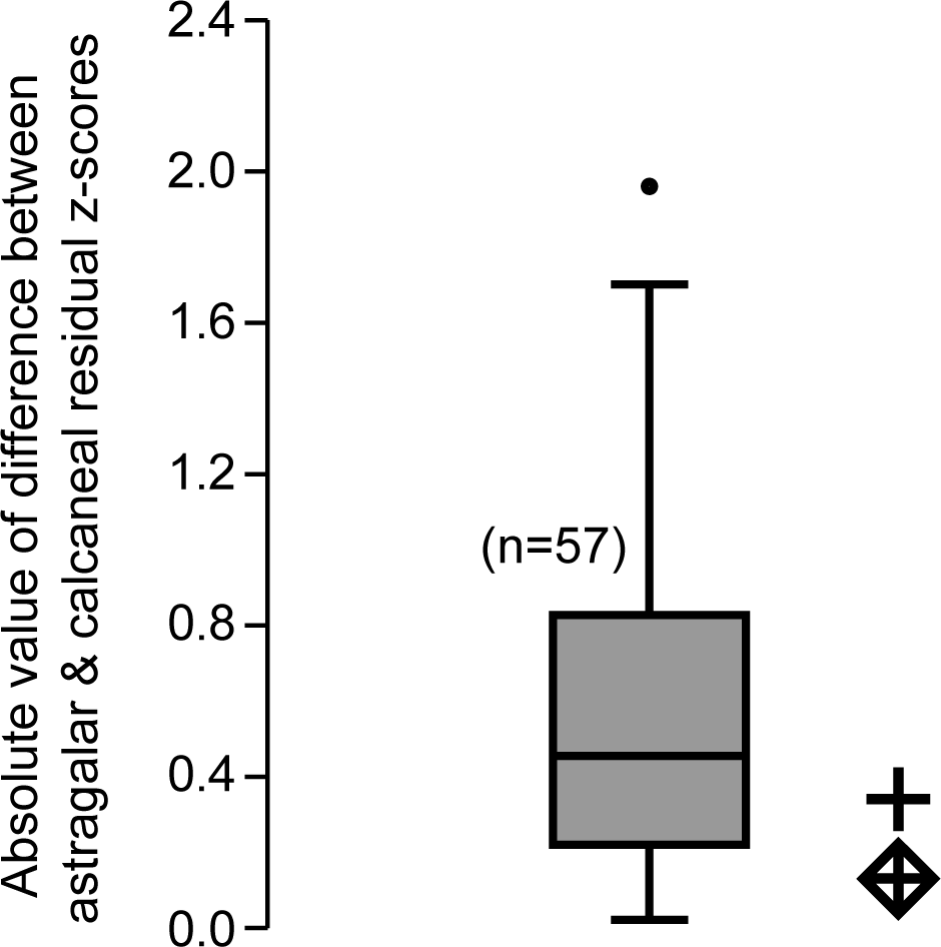
Box plot of *z*-score-standardized differences in residuals. Values plotted are the absolute difference between the z-score-standardized residual of m2 size to astragalus size and the residual of m2 size to calcaneus size of a given taxon. We noticed that for most taxa, the m2 residual generated by the calcaneus was proportional to the m2 residual generated by the astragalus. The comparative sample includes all specimens for which both astragali and calcanei could be compared with tooth size (both fossil and extant) for a total *n* = 57 species. As in other plots, the diamond enclosed cross uses the molar measurements of *Caenopithecus*, while the regular cross uses those of *Leptadapis priscus*. Because the fossils under scrutiny plot in the observed range, this test does not refute the hypothesis that both bones belong to a single species.

The comparison of absolute values of *z*-score differences between calcaneal and astragalar regression residuals helps to address the last concern, though it cannot completely resolve it (Fig. 3). Computing this value for 58 taxa (i.e., all those species with data available for both bones in the original regression, including those of adapines), we find the average difference between *z*-score converted residuals is about 0.6 standard deviation units, with a standard deviation of 0.42 units, and a full range from 0.017–1.96. Whether both fossils are treated as *C. lemuroides*, or both are treated as *L. priscus*, the residual differences do not reject the hypothesis that these bones belong to a single taxon. It also leads to the expectation that if, in fact, one bone represents *C. lemuroides* and the other represents *L. priscus*, then these animals would have had very similarly-sized tarsals overall, despite smaller teeth in the latter.

The last relevant observation emerging from these analyses is that the residual values of putative close relatives of *Caenopithecus* (*Europolemur klatti* (Thalmann, 1994) and *Afradapis* (Seiffert et al., 2009)) are not only positive, but are well above the general adapiform means (Fig. 2). The closest relatives of *L. priscus* in the sample are the adapines *Adapis parisiensis* and *Leptadapis magnus*. For the astragalus, both of those taxa still exhibit a positive residual in contrast to the *L. priscus* attribution, and *A. parisiensis* exhibits a value greater than the adapiform mean. For the calcaneus, *Adapis* also has a positive residual, though it is on the same side of the adapiform mean as the *L. priscus* residual. *L. magnus* has a slightly negative residual, putting it in closer proximity to the *L. priscus* residual. Higher than average positive residuals are therefore expected for caenopithecine tarsal-dental comparisons (meaning that caenopithecines are expected to have larger M_2_s relative to tarsal size than the average adapiform), while neutral to somewhat positive residuals (that are slightly below the average for adapiforms) are expected for adapine tarsal-dental comparisons. This makes sense given what is known about the folivorous caenopithecine *Afradapis* (Seiffert et al., 2009; Seiffert et al., 2010) because primate folivores are expected to have large postcanine teeth relative to body mass (e.g., Kay, 1975; Scott, 2011). Previous analyses of shearing quotients indicate that *Caenopithecus* was very likely folivorous (Kay et al., 2004), and we obtained the same result (Appendix S4, Fig. S1; see dental topographic variables for *Caenopithecus* in Table 3) using the dental topographic comparative framework employed by Seiffert et al. (2010) (though we note that *Caenopithecus* plots close to *Prolemur simus*, which was grouped with folivores in our analysis but is technically a bamboo specialist). As stated above, primate species with folivorous/fibrous diets are expected to have relatively large teeth, and thus large positive residuals of tooth size from tarsal size. Of the candidate species that the tarsals might belong to, *C. lemuroides* residuals meet this expectation, but *L. priscus* does not meet the expectation of neutral to slightly positive residuals based on the data available from other adapines.

Finally, on the basis of the overall morphological pattern, NMB En.270 is more similar to the astragalus of the caenopithecine *Afradapis* than it is to those of adapines. *C. lemuroides* has been placed as the sister taxon of *Afradapis* to the exclusion of all other living and extinct primates in the phylogenetic analysis of Seiffert et al. (2009) (and all later analyses that augmented that matrix), as well as in analysis of a larger character matrix that was independently constructed by Ni et al. (2013). More details on these similarities are presented below. The morphology of NMB En.270 is therefore certainly phylogenetically consistent with attribution to *C. lemuroides*, given what is known about its currently recognized sister taxon. *L. priscus* has been placed as the sister taxon to *L. magnus* and *Adapis* based on dental data (Boyer et al., 2015b), and the latter taxa also show a number of similar morphological features of the astragalus and calcaneus. If the Egerkingen tarsals belonged to *L. priscus*, we would expect the morphology to reflect an antecedent condition, or similar specializations, to those of *Adapis* or *Leptadapis*; instead the tarsal specimens exhibit some unusual specializations that are not expressed in these taxa.

In light of all the foregoing—i.e., on the combined basis of abundance, size (including the higher than average positive residuals for the *Caenopithecus* tarsal-dental comparisons, which fit expectations for a folivorous caenopithecine, but not an adapine), and morphology—we consider the most parsimonious attribution of the Egerkingen tarsals to be to *C. lemuroides* rather than *L. priscus*.

### Body mass estimates

Using the prediction equations published by Yapuncich, Gladman & Boyer (2015) astragalar ectal facet area (AEFa) and calcaneal ectal facet area (CEFa) from NMB En.270 and NMB Eh 719, respectively, returned mean estimates of 1,663 g (AEFa; 95% PI = 659–4,196 g) and 2,217 g (CEFa; 95% PI = 1,098–4,476 g) using their “strepsirrhine” equation; 2,023 g (AEFa; 95% PI = 342–11,984 g) and 2,104 g (CEFa; 95% PI = 609–7,268 g) using their “lorisiform” equation; and 2,962 g (CEFa; 95% PI = 1,392–6,305 g) and 1,964 g (AEFa; 95% PI = 556–6,929 g) using their “lorisid” equation.

### Description of the *Caenopithecus* tarsals

**Figure 4:**
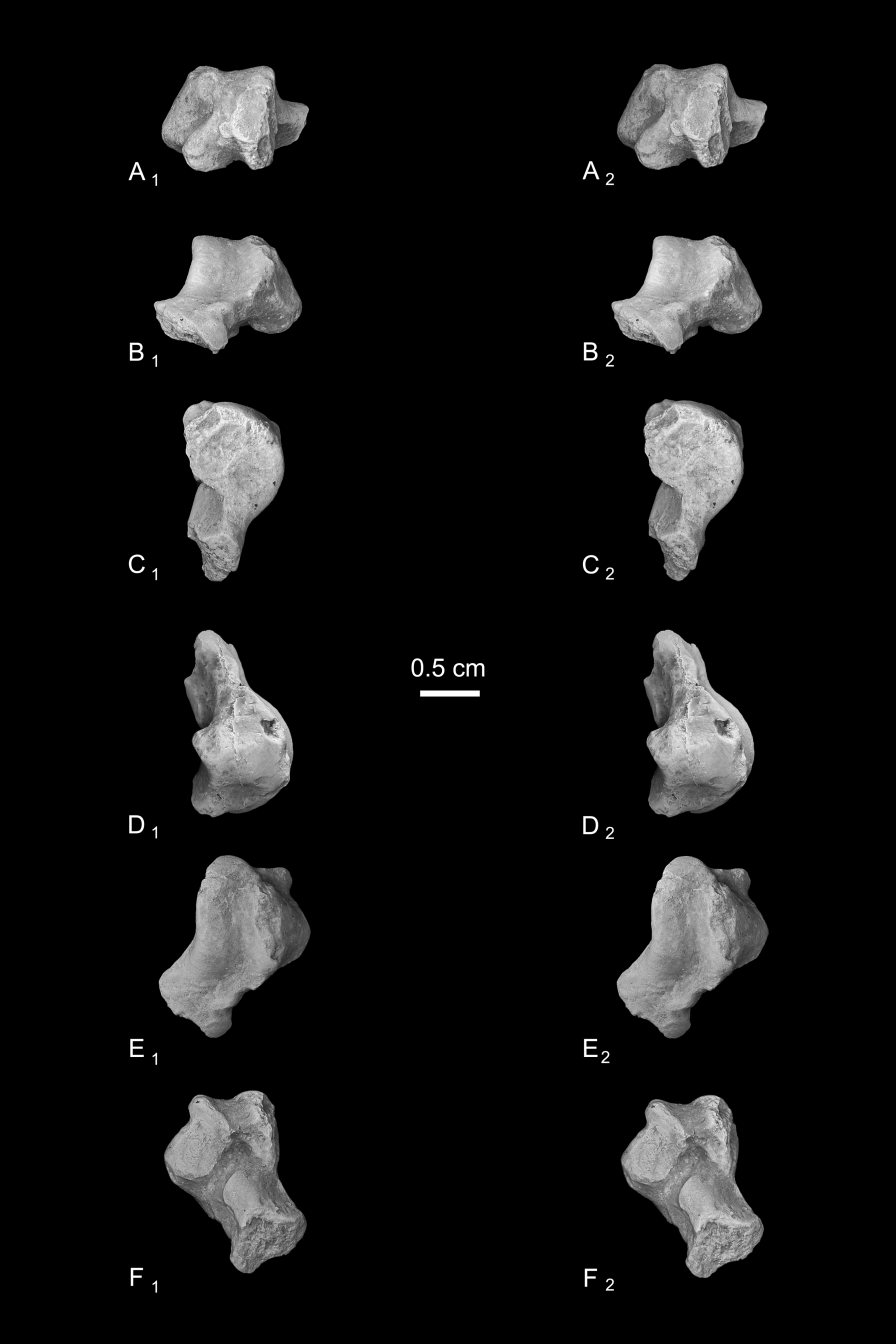
Stereopair images of NMB En.270, left astragalus from Egerkingen (probably fissure *γ*) attributed here to *Caenopithecus lemuroides*. (A) proximal, (B) distal, (C) medial, (D) lateral, (E) dorsal, and (F) plantar views.

Astragalus (NMB En.270, Fig. 4). The astragalus is largely complete, but is abraded along the lateral trochlear rim, the most proximal and medial aspect of the body (the medial tubercle buttressing the groove for the flexor fibularis tendon), and probably (but less clearly) the most distal and lateral aspect of the ectal facet and fibular facets, where those two facets typically meet. The specimen is also missing most of the head and the navicular facet.

In medial view the astragalar body is dorsoplantarly tall, with a medial trochlear rim that has a small radius of curvature (i.e., it is tightly curved for its proximodistal length). The lateral tibial facet (trochlea) bears a shallow sulcus between the medial and lateral rims, which are of about the same height in proximal view. In dorsal view, the lateral tibial facet has a fairly straight medial border along the body (i.e., excluding the distal extension that is confluent with the medial tibial facet), but the lateral border is rounded and tapers strongly toward the proximal and medial aspect of the body; the lateral tibial facet is thus distinctly “v”-shaped in proximal view, and the proximal tapering of the facet allows for a capacious groove for the tendon of the flexor fibularis muscle, which is situated lateral to the lateral tibial facet, as in all known adapiforms and crown strepsirrhines (Beard et al., 1988). Unlike most other primates, this groove extends well onto the plantar aspect of the body and is buttressed laterally by a thick and laterally projecting flange, which also supports the most proximal aspect of the ectal facet, and presumably served as an attachment site for the posterior astragalo-fibular ligament. There is no hint of a posterior astragalar shelf or a superior astragalar foramen.

The fibular facet is proximodistally convex and quite large, covering most of the lateral aspect of the astragalar body in lateral view. The fibular facet slopes laterally from the lateral tibial facet at an angle of 112° (using the measurement protocol described by Boyer & Seiffert, 2013). The medial tibial facet is large, taking up more than half of the medial surface of the astragalar body and extending all the way to its plantar surface, as in many other “prosimian” primates (Boyer et al., 2015b). The facet continues distally, becoming dorsoplantarly shorter as it curves onto the medial surface of the astragalar neck. There is a dorsoplantarly tall but shallow bean-shaped fossa proximal to the medial tibial facet, presumably for attachment of the posterior tibioastragalar portion of the deltoid ligament. The articular area of the medial tibial facet measures 25.7 mm^2^. The medial tubercle buttressing the groove for the flexor fibularis tendon is abraded proximally, but the plantar border of the medial wall of the body clearly extends far plantarly as a protruding ridge, which terminates just distal to the most proximal extension of the sustentacular facet. This plantar projection forms the medial half of the deep trochlear groove for the flexor fibular tendon as it passes plantarly around the astragalus, and clearly contributes to the perception that the astragalar body is tall.

We estimate that the astragalar neck meets the body at approximately a 33° angle. Judging from the shape of the astragalar neck along its broken surface, the navicular facet likely would have been mediolaterally quite broad relative to its dorsoplantar height. A small part of the navicular facet is preserved on the lateral aspect of the neck, revealing that that part of the facet, at least, was clearly convex. On the plantar surface, the proximal part of the sustentacular facet is preserved, and is strikingly convex, with articular surface extending medially and laterally away from the plantar apex of the facet. There is no concave extension of the sustentacular facet along its proximal margin, as occurs in some primates. The facet’s proximolateral border is well-defined, but the proximomedial border is not, sloping gradually toward the sulcus that separates the facet from the plantar ridge on the body’s medial wall. An elongate tubercle is present on the dorsal surface of the neck, presumably for attachment of the astragalar-ectocuneiform ligament.

The ectal facet is roughly rectangular in plantar view, with a laterally projecting proximal part where the facet extends out onto the lateral tubercle for the flexor fibularis groove. The medial margin of the ectal facet is well-defined and projects plantarly, forming the lateral wall of the deep flexor fibularis groove. The area of the ectal facet is 20.6 mm^2^. See Table 2 for astragalar measurements taken on the specimen that follow the methods of Gebo et al. (2001).

**Table 2:** Measurements of the Egerkingen tarsals, following Gebo et al. (2001).

Measurement	Specimen		
	NMB En.268	NMB En.269	NMB Eh 719
“Calcaneal length” (CalL)	22.25		20.74
“Distal calcaneal length” (DistL)			7.76
“Posterior calcaneal facet length” (PcfL, = ectal facet of this study)		7.54	6.87
“Posterior calcaneal facet width” (PcfW)	2.61		3.77
“Heel length” (HeelL)	8.98		6.24
“Calcaneal width” (CalW)			10.11
“Calcaneocuboid height” (CubHt)			4.17
“Calcaneocuboid width” (CubW)			6.51
CalW/CalL			0.49
DistL/CalL			0.37
PcfL/CalL			0.33
HeelL/CalL	0.40		0.30
PcfL/HeelL			1.10
PcfW/PcfL			0.55
CubW/CubHt			1.56

	NMB En.270
“Talar neck angle” (TNECKANGLE, in degrees, estimated)	33
“Trochlear length” (TRL)	9.33
“Midtrochlear width” (MTRW)	6.25
“Talar width” (TW)	9.28
MTRW/TRL	0.67

**Table 3:** Relief index (RFI) and orientation patch count (OPC) values for *P*_4_ and M_2_ of *Caenopithecus lemuroides*.

Specimen	Species	Locus	2D area	3D area	RFI	OPC
NMB no number	*Caenopithecus lemuroides*	p4	15.77	43.18	0.50	35.50
NMB no number	*Caenopithecus lemuroides*	m2	25.96	67.60	0.48	68.00
NMB Eh 396	*Caenopithecus lemuroides*	m2	25.89	67.24	0.48	54.13
NMB Eh 735	*Caenopithecus lemuroides*	m2	26.05	71.03	0.50	62.63
NMB Eh 600	*Caenopithecus lemuroides*	p4	16.10	38.86	0.44	28.63
NMB Eh 597	*Caenopithecus lemuroides*	p4	14.59	41.40	0.52	36.88
NMB Eh 597	*Caenopithecus lemuroides*	m2	25.24	68.45	0.50	56.25

**Figure 5:**
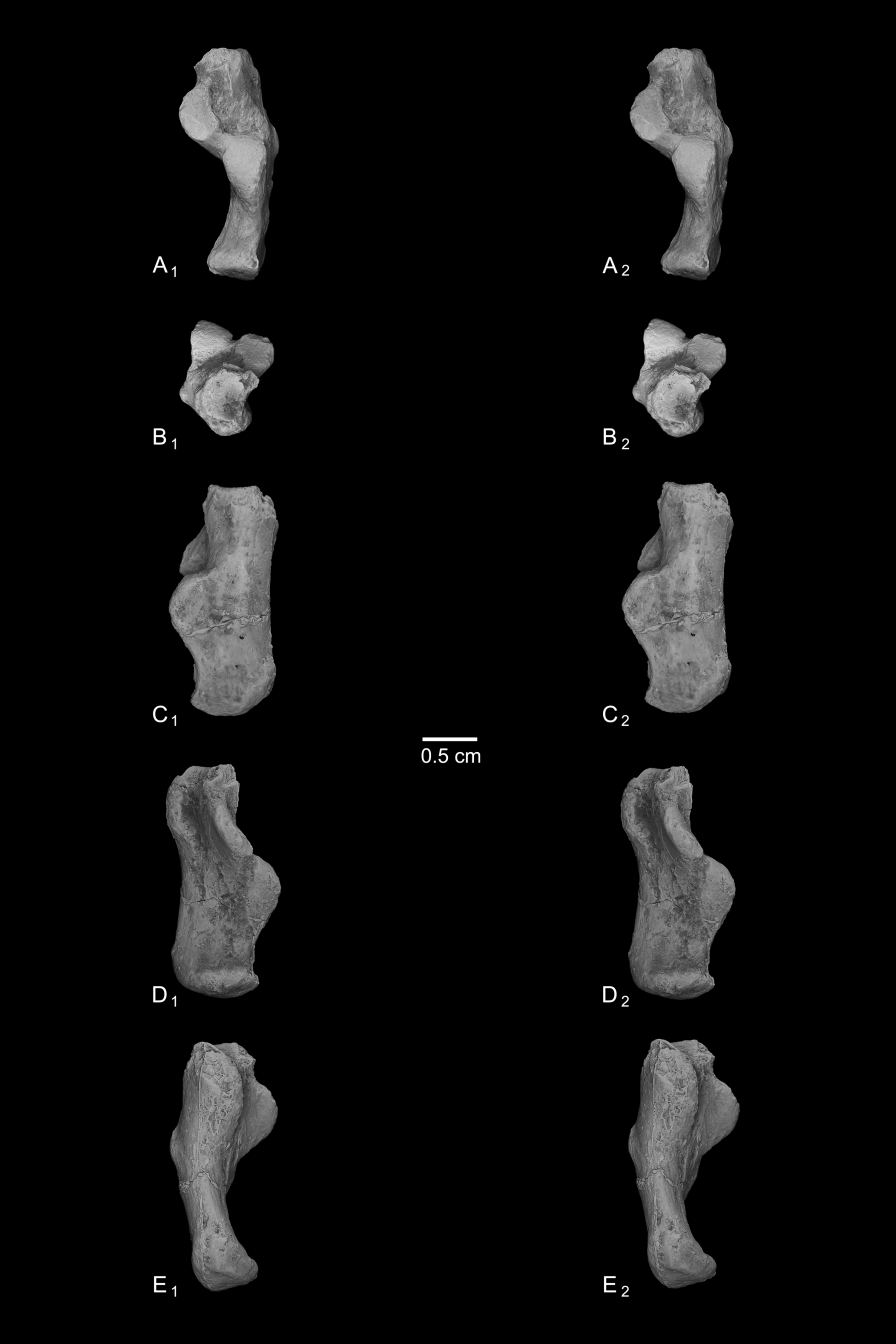
Stereopair images of NMB Eh 719, right calcaneus from Egerkingen fissure *γ*, attributed here to *Caenopithecus lemuroides*. (A) dorsal, (B) distal, (C) lateral, (D) medial, and (E) plantar views.

**Figure 6:**
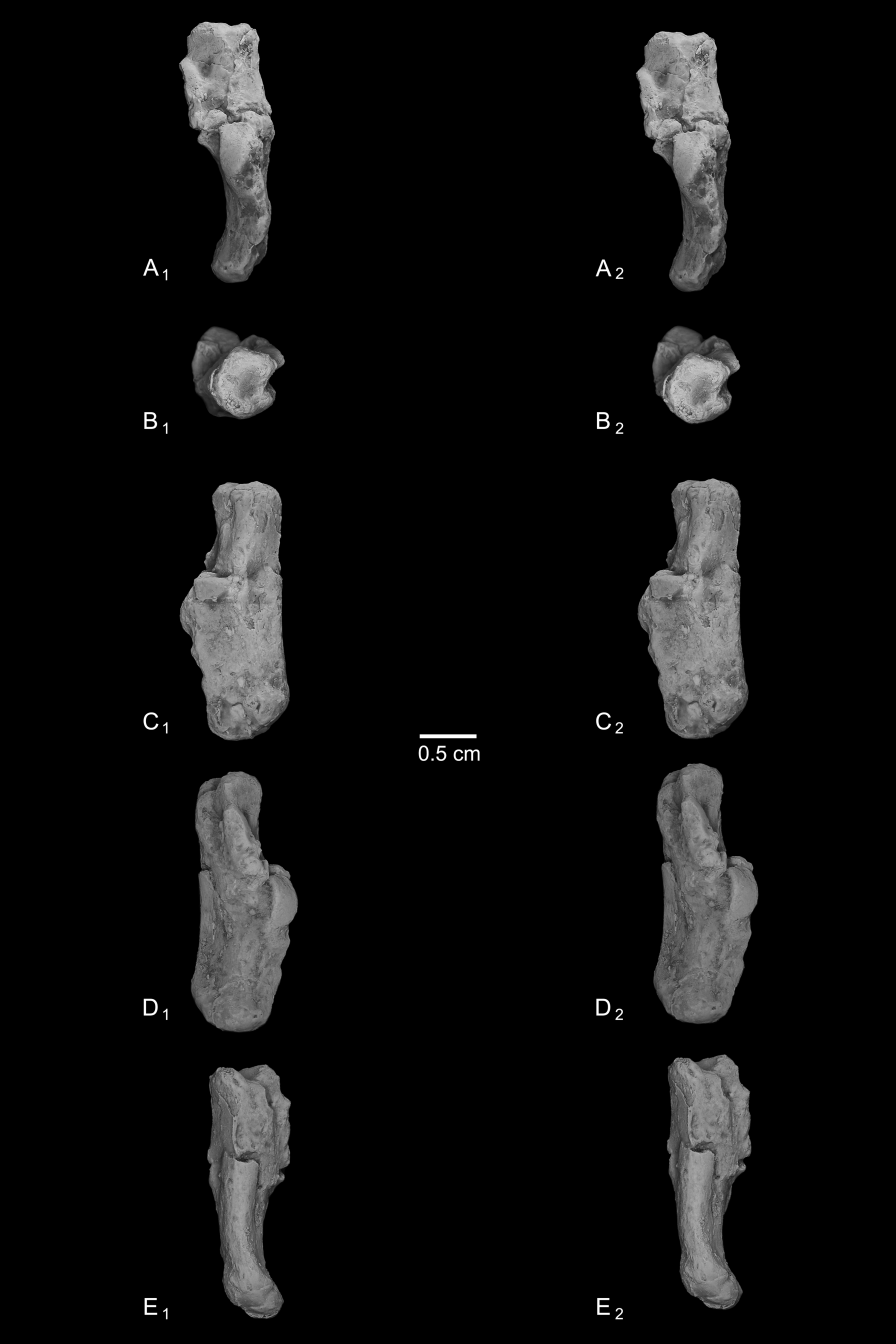
Stereopair images of NMB En.268, right calcaneus from Egerkingen (probably fissure *γ*) attributed here to *Caenopithecus lemuroides*. In (A) dorsal, (B) distal, (C) lateral, (D) medial, and (E) plantar views.

**Figure 7:**
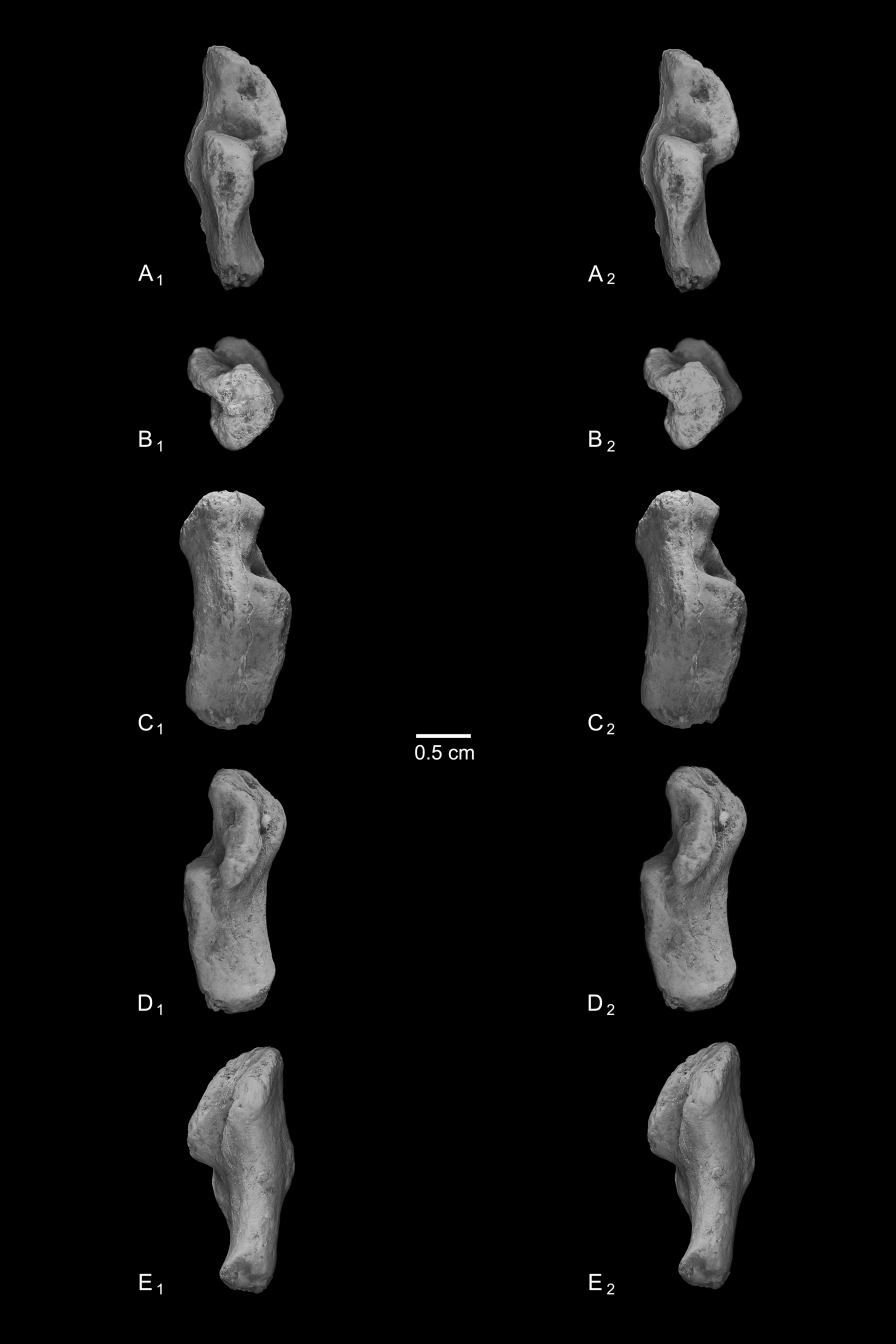
Stereopair images of NMB En.269, left calcaneus from Egerkingen (probably fissure *γ*) attributed here to *Caenopithecus lemuroides*. In (A) dorsal, (B) distal, (C) lateral, (D) medial, and (E) plantar views.

Calcanei (NMB Eh 719 (Fig. 5), NMB En.268 (Fig. 6), and NMB En.269 (Fig. 7)). Three primate calcanei have been identified in the Egerkingen collections. As already discussed, the three calcanei are of approximately the same size and, despite some differences, conform to a unique morphological pattern. Unless otherwise mentioned, the following description is based largely on NMB Eh 719, which is both the best-preserved calcaneus, and the only specimen that is known to be from fissure *γ* based on available records (Fig. 5). Of the other two calcanei, NMB En.268 (Fig. 6) is missing the medial part of the sustentaculum and has a large crack passing mediolaterally through the sustentaculum and ectal facet, leading to displacement of the two halves of the bone relative to each other; it is also missing most of the cortical bone on the lateral surface of the calcaneal tuber. The medial aspect of the sustentaculum of NMB En.269 (Fig. 7) is also missing, and the bone is badly abraded all along the medial and plantar surface, including the cuboid facet. In dorsal view, most of the lateral part of the ectal facet of NMB En.269 is missing, and the proximal aspect of the calcaneal tuber is badly damaged.

The distal segment of NMB Eh 719 makes up approximately 37% of total calcaneal length and is not dorsally “flexed” relative to the proximal segment (see Gladman et al., 2013). The cuboid facet of NMB Eh 719 is damaged along its dorsomedial and plantar surface, but it is clear that the articular surface is “fan”-shaped, with a long axis that is oriented obliquely with respect to the dorso-plantar axis of the calcaneal body. There is a distinct concavity along the medial and plantar surface of the facet for articulation with a proximally projecting process of the cuboid. Medial and plantar to this articular pit is a proximodistally elongate distal calcaneal tubercle (best developed in NMB Eh 719 and NMB En.269). The cuboid facet and its margins are best preserved on NMB En.268, and this specimen confirms that the sustentacular facet does not extend to the distal end of the calcaneus, that there is no secondary sustentacular facet, and that there is no facet for the navicular distal to the sustentacular facet. At its broadest point (i.e., at the most medial projection of the sustentacular shelf), NMB Eh 719 is about 48% as wide as the calcaneus is long. The sustentaculum does not have a deep groove for the passage of the flexor fibularis tendon, but rather is quite flat—particularly that of NMB Eh 719, but somewhat less so in NMB En.268. This condition is surely correlated with the dorsolateral orientation of the entire sustentacular shelf, which is best appreciated in distal view (Figs. 5D, 6D and 7D). In dorsal view the sustentacular facet of NMB Eh 719 is proximodistally elongate, bean-shaped, and bears a gentle lateral concavity; it tapers distally and plantarly to meet the body of the calcaneus (the facet is, however, broken along its proximal and medial margin). The proximal margin of the sustentaculum does not bear a convex articular surface for a proximal extension of the astragalar sustentacular facet, as occurs in some other Paleogene primates. The ectal facet is about 55% as wide as it is long, and tapers proximally but remains quite broad distally. The facet is tightly curved in all specimens, though on NMB En.268 this condition is obscured somewhat by breakage and displacement of the distal part of the facet. The dorsal surface of the facet does not project out laterally above the lateral border of the calcaneus, as occurs in some primates that consequently bear a concave surface inferolateral to the ectal facet. The peroneal tubercle is placed at approximately the distal margin of the ectal facet, is longer (proximodistally) than it is high (dorsoplantarly), and does not project far laterally; it is not elongate and shelf-like as in some other Paleogene primates. The lateral wall of the distal segment in *C. lemuroides* appears to be medially oriented with respect to the proximal segment, but this might also be interpreted as a consequence of medial bowing of the calcaneal tuber with respect to the distal segment. The calcaneal tuber bears distinct rugosities not only along the dorsal surface but also along the medial margin, further contributing to its medially bowed appearance. See Table 2 for calcaneal measurements taken on the specimens that follow the methods of Gebo et al. (2001).

### Comparisons with other strepsirrhine astragali

Given *Caenopithecus*’ well-supported phylogenetic placement among “adapiforms”—an assemblage of fossil primates that are basally diverging within the order and that may or may not be paraphyletic with respect to crown strepsirrhines (see phylogenetic results), we restrict our comparisons largely to these taxa, specifically adapines (Dagosto, 1983; Decker & Szalay, 1974), notharctines (Gebo, 1988; Gebo, Dagosto & Rose, 1991), asiadapines (Rose et al., 2009), *Anchomomys* (Moyà-Solà & Köhler, 1993; Moyà-Solà et al., 2011), *Azibius* (Marivaux et al., 2011), and *Djebelemur* (Marivaux et al., 2013). We also make comparisons based on figures of isolated specimens that have been attributed to *Europolemur klatti* (Thalmann, 1994) and figures and half-casts of specimens that have been attributed to *Kyitchaungia takaii* (Beard et al., 2007) from the middle Eocene of Germany and Burma, respectively. Beard et al. (2007) consider *Kyitchaungia* to be a sivaladapid; if correct, the specimens might be the only known tarsal elements from that clade (though we note that, on the basis of our comparisons of M_2_ size to tarsal dimensions, these specimens could also be attributable to the amphipithecid *Myanmarpithecus yarshensis*—a possibility that Beard et al. (2007) did not explicitly consider). Here we make comparisons with the astragalus (NMMP 59) and best-preserved calcaneus (NMMP 58) that Beard et al. (2007) attribute to *Kyitchaungia*. We also make comparisons with the distal calcaneus that is part of the problematic partial skeleton NMMP 20, from the late middle Eocene of Myanmar; this partial skeleton is either that of an amphipithecid, as originally suggested on the basis of the dental remains known from Sabapondaung kyitchaung locality (Ciochon et al., 2001), or is a sivaladapid (dental remains of which have not been found at the locality; Beard et al., 2007). In addition, we discuss similarities that the *Caenopithecus* tarsals share with those of lorisids, the subfossil indrioid *Babakotia*, and some other extant strepsirrhines, largely because of their importance for functional interpretation. Note that the same comparisons would also be appropriate if the Egerkingen tarsals actually belong to *L. priscus*.

**Figure 8:**
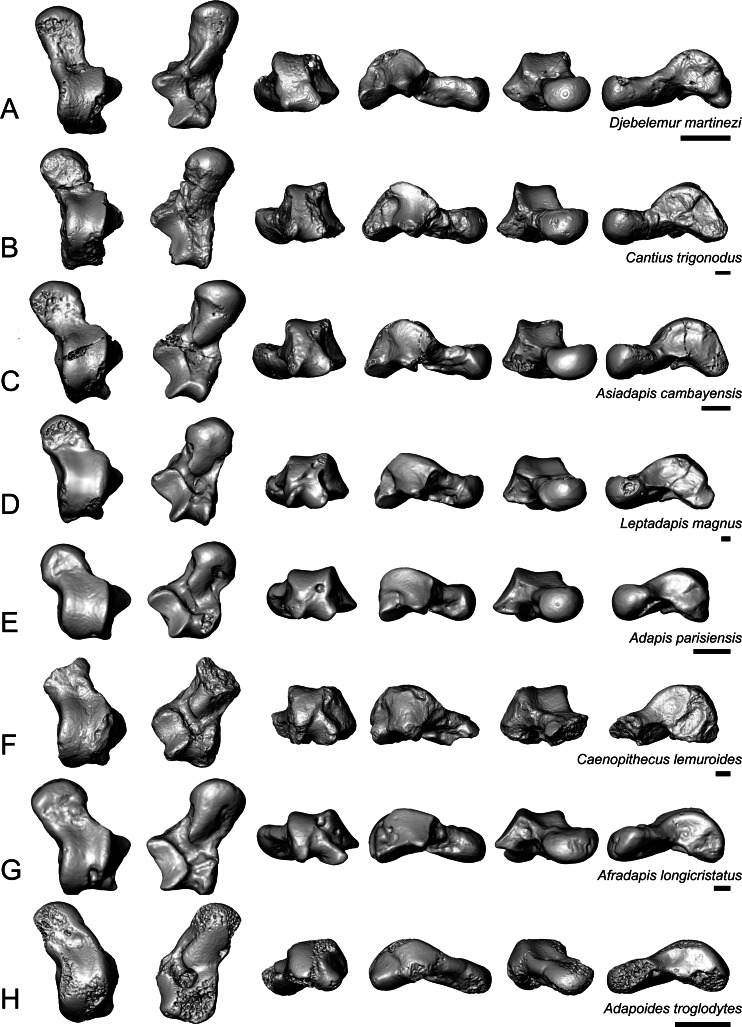
Astragali of other fossil strepsirrhines compared to NMB En.270, attributed here to *Caenopithecus lemuroides*. (A) *Djebelemur martinezi* (CBI-1-545), from the early or middle Eocene of Tunisia; (B) *Cantius trigonodus* (USGS 21832), from the early Eocene of the USA; (C) *Asiadapis cambayensis* (GU 747), from the early Eocene of India; (D) *Leptadapis magnus* (MNHN QU 11001), from the late Eocene of France; (E) *Adapis parisiensis* (ECA 1379), from the late Eocene of France; (F) *Caenopithecus lemuroides* (NMB En.270, reversed); (G) *Afradapis longicristatus* (DPC 21445C), from the late Eocene of Egypt; (H) *Adapoides troglodytes* (IVPP V13016, reversed), from the middle Eocene of China. Views in, from left to right, dorsal, plantar, proximal, lateral, distal, and medial views. Scale bars are equal to 1 mm.

**Figure 9:**
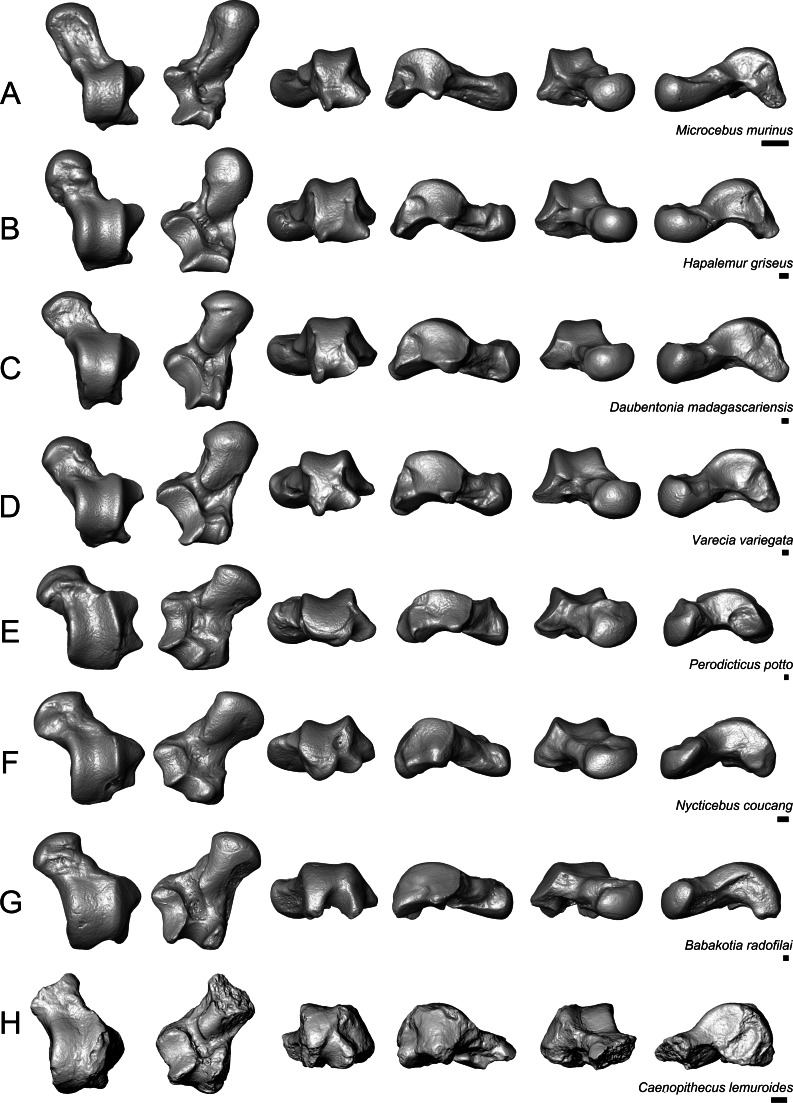
Astragali of extant and subfossil strepsirrhines compared to NMB En.270, attributed here to *Caenopithecus lemuroides.* (A) *Microcebus murinus* (AMNH 174430); (B) *Hapalemur griseus* (AMNH 170680); (C) *Daubentonia madagascariensis* (AMNH 119694); (D) *Varecia variegata* (AMNH 201384); (E) *Perodicticus potto* (AMNH 184579); (F) *Nycticebus coucang* (AMNH 212953); (G) *Babakotia radofilai* (DPC 11000); (H) *Caenopithecus lemuroides* (NMB En.270, reversed). Views in, from left to right, dorsal, plantar, proximal, lateral, distal, and medial views. Scale bars are equal to 1 mm.

*Caenopithecus* shows a unique mix of astragalar features, some of which are seen in *Adapis* and *Afradapis*, and others of which are more similar to conditions seen in *Leptadapis* and notharctines. The astragalar body is relatively tall (dorsoplantarly) when compared with those of *Adapis* (Fig. 8E), *Afradapis* (Fig. 8G), lorises (Figs. 9E and 9F), and *Babakotia* (Fig. 9G). The plantar aspect of the proximal portion of the astragalar body is not preserved in *Adapoides* (Fig. 8H), but the remaining morphology suggests that the astragalar body was probably fairly low, perhaps as in *Afradapis*. Among other stem strepsirrhines, relatively tall astragalar bodies are also seen in *Leptadapis* (Fig. 8D), notharctines such as *Cantius* (Fig. 8B), asiadapines (Fig. 8C), *Anchomomys*, *Azibius*, *Djebelemur* (Fig. 8A), and NMMP 59. The height of *Babakotia*’s astragalar body in medial view is exaggerated by its tall plantarly-projecting medial process buttressing the groove for the tendon of flexor fibularis (Fig. 9G).

The very well-defined triangular proximal extension of the lateral tibial facet is most like those of *Afradapis* and *Babakotia* (Figs. 8G and 9H). Other living and extinct strepsirrhines have facets that taper posteriorly, but they are not so distinctly set off from the flexor fibularis groove. In *Caenopithecus* that groove is deeply excavated and extends onto the plantar aspect of the body, as in adapines (Fugs. 8D and 8E), *Afradapis* (Fig. 8G), *Babakotia* (Fig. 9G), lorises (Figs. 9E and 9F), and, to a lesser extent, *Varecia* (Fig. 9D). In *Afradapis*, *Caenopithecus*, *Babakotia*, and some lorises, the proximomedial margin of the ectal facet projects distinctly plantar to the groove, forming its lateral wall. The plantar ridge forming the medial wall of this groove in *Caenopithecus* is also well-developed in *Leptadapis* (see MNHN QU 11001, Fig. 8D) and *Babakotia*, but is not as distinct in *Adapis* and *Afradapis*. This plantar projection clearly contributes to the perception that *Caenopithecus* and *Leptadapis* have tall astragalar bodies, but in medial view the neck and medial tibial facet of *Caenopithecus* are actually more similar to those of *Afradapis*, aside from the strongly projecting plantar ridge.

The medial tibial facet of *Caenopithecus* is dorsoplantarly deep as in all other adapiforms, but specifically resembles that of *Afradapis* in becoming dorsoplantarly shorter as it curves onto the medial surface of the astragalar neck; this similarity is surely also due to the relatively long astragalar necks of *Afradapis* and *Caenopithecus* when compared to those of adapines. The ln of the square root of medial tibial facet area relative to ectal facet area is 0.11, which is higher than that of any Paleogene primate. The only extant and subfossil primates that equal or exceed this value (i.e., that have an equal, or higher, ratio of medial tibial facet area relative to ectal facet area) are indriids, some lemurids, galagids, some lorisids (*Arctocebus* and *Nycticebus*), and *Palaeopropithecus*.

The complete absence of a posterior trochlear shelf also characterizes *Adapis*, *Adapoides*, *Afradapis*, *Babakotia*, and lorises; such “shelves” are present, to varying degrees, in notharctines, *Anchomomys*, *Djebelemur*, and some extant strepsirrhines, and Beard et al. (2007) suggest that one was probably present on NMMP 59. In medial view *Leptadapis* appears to bear a posterior trochlear shelf, but this posterior bulge is composed solely of the plantarly projecting ridge buttressing the flexor fibular groove, and there is no shelf extending across the proximal and plantar aspect of the body as in some other adapiforms and crown strepsirrhines. The absence of a superior astragalar foramen differs from the condition in *Afradapis*, *Leptadapis*, and some specimens of *Adapis*; this foramen also occurs variably in lorisids, but not in any other extant primates of which we are aware.

The fibular facet angle of 112° is slightly higher than those that have been calculated for *Leptadapis* (104–109°, *n* = 3), but is within the range of *Adapis* (106–114°, *n* = 8) and *Babakotia* (98–125°, *n* = 3), close to that of *Djebelemur* (113°), and lower than that of the single *Afradapis* specimen that is currently known (116°) (Boyer & Seiffert, 2013). Other adapiforms have lower values, for instance basal *Cantius* (94°−103°), *Pelycodus* (96°−108°), *Asiadapis* (100°), *Marcgodinotius* (106°−110°), and *Anchomomys* (105–111°).

The astragalar neck meets the body at approximately a 33° angle, similar to that of *Afradapis* and the values reported by Gebo et al. (2001) for *Adapis* and *Leptadapis*, but higher than the values that they reported for notharctines aside from *Notharctus tenebrosus* (35°). Among smaller stem strepsirrhines, astragalar neck angle is also relatively low—29° in *Asiadapis*, 20–32° in *Marcgodinotius*, 19° in *Anchomomys*, 17–18° in *Azibius*, 20° in *Djebelemur*, and 26° in NMMP 59 . The possible *Europolemur klatti* specimen (CeIV-2852) also appears to have a low neck angle, certainly lower than those of *Afradapis* or *Caenopithecus*. Lorises and *Babakotia* have particularly high neck angles.

The elongate tubercle on the dorsal surface of the neck for the astragalar-ectocuneiform ligament is also well-developed in *Adapoides*, *Afradapis*, *Babakotia*, lorises, many notharctines, and apparently on *Europolemur*, but is not clearly expressed to the same degree in adapines.

In many omomyiforms, asiadapines, adapines, and *Adapoides* there is a concave proximal extension of the sustentacular facet that would form a sort of locking mechanism with a posteriorly convex proximal extension of the sustentacular facet on the calcaneus (Boyer, Seiffert & Simons, 2010); in contrast, there is no distinct articular surface for such a proximal extension in either *Caenopithecus* or *Afradapis*. *Caenopithecus* is fairly unique among adapiforms in having a strongly mediolaterally convex proximal portion of the sustentacular facet.

### Multivariate morphometric analysis of primate astragali

**Figure 10:**
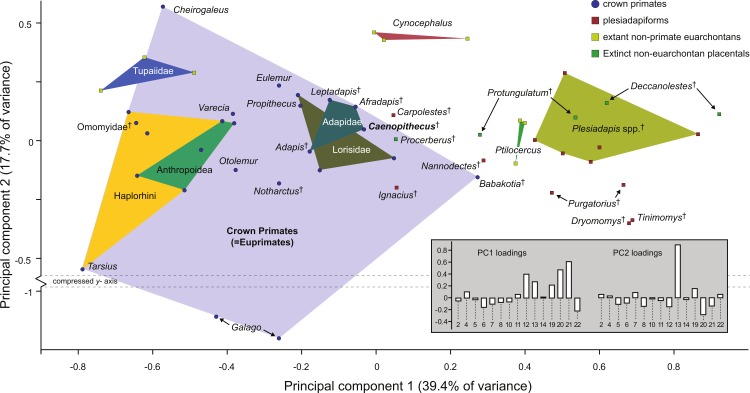
Principal components analysis of astragalar shape variables and angles. First two principal component axes, accounting for 57.1% of the overall variance, based on the reduced dataset of 15 astragalar measurements that could be taken on NMB En.270 (loadings for each variable on PC1 and PC2 are provided in the lower right hand corner). Note that *Caenopithecus* falls close to *Afradapis*, and to adapines and lorisids. The suspensory subfossil palaeopropithecid “sloth lemur” *Babakotia* expands the crown primate morphospace considerably, driven largely by extreme expression of features that also influence *Caenopithecus*’s positive score on PC1—i.e., a particularly long and wide flexor fibularis groove (variables 12 and 13) and a high angle between the medial tibial facet and fibular facet (variable 20).

Along principal component one (which explains 39.4% of the variance) *Caenopithecus* falls close to *Afradapis*, and, among extant primates sampled, overlaps solely with the morphospace occupied by lorisids (Fig. 10). Among non-primates, *Caenopithecus*’ PC1 score is close to that of some *Cynocephalus* individuals, as well as the extinct carpolestid *Carpolestes* and paromomyid *Ignacius*. The strongest loadings along this axis are for variables 12 (flexor fibularis groove width), 20 (angle between fibular facet and medial tibial facet), and 21 (angle between medial and lateral tibial facets), all of which are positive. The positions of *Caenopithecus*, *Afradapis*, adapines, *Babakotia*, and lorisids along PC1 reflect the fact that, relative to other crown primates, they have broad flexor fibularis grooves, high (obtuse) angles between the fibular facet and the medial tibial facet, and low (acute) angles between the medial and lateral tibial facets. Principal component 2 explains 17.7% of the variance and does not clearly separate extant primates along functional lines; this component is dominated by a strong positive loading for variable 13 (flexor fibularis groove proximodistal length). Again, *Caenopithecus* overlaps with the adapine-caenopithecine and lorisid morphospaces along this axis (but also that of many other primates and non-primates). Overall, among extant primates that might guide functional interpretation, the clearest phenetic affinities along the principal two axes are with the cautious and slow-climbing lorisids. Importantly, though, on the far negative range of the lorisid morphospace there is near-overlap with the extant large-bodied acrobatic leaper *Propithecus*, presumably reflecting the fact that the reduced sample of variables that can be measured on the *Caenopithecus* astragalus does not adequately capture all of the functionally-informative morphology provided by this element (though we note that *Propithecus* is also capable of hind limb suspension in addition to acrobatic leaping).

### Flexor fibularis groove depth

**Figure 11:**
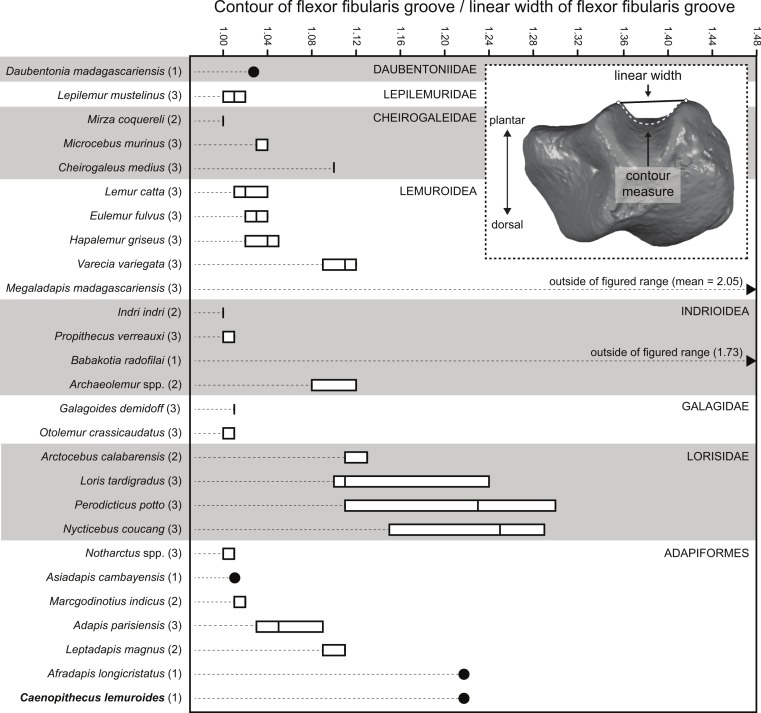
Quantification of flexor fibularis groove depth on the plantar surface of the astragalus. Inset image shows the orientation of the astragalus for simultaneous measurement of linear width of the flexor fibularis groove, and the contour measure of the groove (taken in Geomagic). As noted in the main text, astragali were oriented with the plantar surface facing upward and in posterior view, such that the point marking the base of the trough of the flexor fibularis groove was aligned with the point marking the plantar apex of the navicular facet or sustentacular facet (whichever was visible in that view). Boxplots show variation within species in the ratio of the contour measurement to the linear measurement; higher numbers are found in taxa with deeper flexor fibularis grooves. Note that the values for *Babakotia* and *Megaladapis* are so extreme that they fall far outside of the figured range.

Quantification of flexor fibularis groove depth among living and extinct strepsirrhines revealed that most extant species have ratios of flexor fibularis groove contour length to flexor fibularis groove linear width of 1 or only slightly higher, indicating that there is very little concavity of the groove along the plantar surface of the astragalus (Fig. 11 and Table 4). This is universally characteristic of the particularly acrobatic grasp-leaping strepsirrhines, such as the galagids *Galagoides* and *Otolemur*, the lemurid *Hapalemur*, the lepilemurid *Lepilemur*, and the indriids *Indri* and *Propithecus*, but is also seen in extant lemurs with more generalized locomotor behavior. The major departures from ratios of 1 are seen in *Cheirogaleus*, *Varecia*, and particularly lorises, which have some of the highest values among extant taxa (of which *Nycticebus* and *Perodicticus* have the highest). The highest values among strepsirrhines, however, were found among the subfossil forms *Babakotia* (ratio of 1.73) and *Megaladapis* (mean ratio of 2.05). *Archaeolemur* also had a relatively high value when compared with those of most extant lemurs (mean of 1.1, close to that of *Varecia*). Among Eocene adapiforms, the phylogenetically basal taxa *Asiadapis*, *Marcgodinotius*, and *Notharctus* had values close to 1, while *Afradapis* and *Caenopithecus* both had values of 1.22, which is higher than the values of all extant strepsirrhines aside from those of some lorises (and note that the value for *Caenopithecus* is a minimum estimate, due to damage to the medial plantar ridge buttressing the groove). The adapines *Adapis* and *Leptadapis* had intermediate values, with means of 1.05 and 1.10, respectively.

**Table 4:** Ratios of flexor fibularis groove contour measures versus flexor fibularis groove linear width in living and extinct strepsirrhines.

Taxon	Specimen	Flexor fibularis groove contour/flexor fibularis groove linear
*Adapis parisiensis*	MaPhQ 1390	1.03
*Adapis parisiensis*	ROS 106	1.05
*Adapis parisiensis*	ROS 2708	1.09
*Afradapis longicristatus*	DPC 21445C	1.22
*Archaeolemur* sp.	DPC 7849	1.08
*Archaeolemur* sp.	DPC 7900	1.12
*Arctocebus calabarensis*	AMNH 207949	1.11
*Arctocebus calabarensis*	AMNH 212576	1.13
*Asiadapis cambayensis*	GU 747	1.01
*Babakotia radofilai*	DPC 11000	1.73
*Caenopithecus lemuroides*	NMB En.270	1.22
*Cheirogaleus medius*	DPC 0142	1.10
*Cheirogaleus medius*	DPC 031	1.10
*Cheirogaleus medius*	DPC 1023	1.10
*Daubentonia madagascariensis*	AMNH 119694	1.03
*Eulemur fulvus albifrons*	AMNH 170708	1.03
*Eulemur fulvus albifrons*	AMNH 170728	1.04
*Eulemur fulvus fulvus*	AMNH 31254	1.02
*Galagoides demidoff*	AMNH 212956	1.01
*Galagoides demidoff*	AMNH 241121	1.01
*Galagoides demidoff*	AMNH 215180	1.01
*Hapalemur griseus*	AMNH 170680	1.02
*Hapalemur griseus*	AMNH 170689	1.04
*Hapalemur griseus*	AMNH 61589	1.05
*Indri indri*	AMNH 208992	1.00
*Indri indri*	AMNH 100504	1.00
*Lemur catta*	AMNH 170739	1.02
*Lemur catta*	AMNH 170740	1.04
*Lemur catta*	AMNH 170765	1.01
*Lepilemur mustelinus*	AMNH 170556	1.01
*Lepilemur mustelinus*	AMNH 170560	1.02
*Lepilemur mustelinus*	AMNH 170565	1.00
*Leptadapis magnus*	NMB QE 261	1.11
*Leptadapis magnus*	NMB QE 496	1.09
*Loris tardigradus*	AMNH 150038	1.24
*Loris tardigradus*	AMNH 165931	1.10
*Loris tardigradus*	AMNH 34257	1.11
*Marcgodinotius indicus*	GU 748	1.02
*Marcgodinotius indicus*	GU 749	1.01
*Megaladapis madagascariensis*	DPC 18936	1.92
*Megaladapis madagascariensis*	DPC 17176	1.81
*Megaladapis madagascariensis*	DPC 7821	2.43
*Microcebus murinus*	AMNH 174428	1.03
*Microcebus murinus*	AMNH 174430	1.04
*Microcebus murinus*	AMNH 174431	1.03
*Mirza coquereli*	DPC 0137	1.00
*Mirza coquereli*	DPC 1139	1.00
*Notharctus* sp.	AMNH 12000	1.01
*Notharctus* sp.	AMNH 11474	1.00
*Notharctus* sp.	AMNH 129382	1.01
*Nycticebus coucang*	AMNH 90381	1.29
*Nycticebus coucang*	AMNH 102027	1.25
*Nycticebus coucang*	AMNH 212953	1.15
*Otolemur crassicaudatus*	AMNH 187364	1.00
*Otolemur crassicaudatus*	AMNH 150413	1.01
*Otolemur crassicaudatus*	AMNH 216240	1.00
*Perodicticus potto*	AMNH 184579	1.23
*Perodicticus potto*	AMNH 269851	1.11
*Perodicticus potto*	AMNH 86898	1.30
*Propithecus verreauxi*	AMNH 170474	1.00
*Propithecus verreauxi*	AMNH 170463	1.01
*Propithecus verreauxi*	AMNH 208991	1.00
*Varecia variegata*	AMNH 201384	1.11
*Varecia variegata*	DPC 049	1.12
*Varecia variegata*	AMNH 100512	1.09

### Comparisons with other strepsirrhine calcanei

In terms of overall morphology and proportions, the calcanei of *C. lemuroides* are similar to those of *Adapis* and *Leptadapis* in having mediolaterally broad ectal facets (relative to proximodistal length), well-developed distal calcaneal tubercles, and “fan”-shaped (rather than strictly ovoid) facets for the articulating cuboid. This *gestalt* similarity to adapines is further supported by automated geometric analysis of calcanei from multiple living and extinct primates (see below). The *C. lemuroides* calcanei also show some striking specializations that, as a complex, clearly set them apart not only from adapines but all other adapiforms—including features such as tightly curved ectal facets that project dorsal to the calcaneal tubers, a convex lateral border of the calcaneus, and sustentacular shelves that are dorsolaterally inclined relative to the mediolateral plane of the ectal facets’ dorsal apices. Outside of adapiforms, some of these features can be found among lorises and *Babakotia*, and these probable convergences help to guide our functional inferences.

Relative elongation of the distal calcaneal segment in *Caenopithecus* (37% of total calcaneal length in NMB Eh 719) is intermediate between the very foreshortened condition exhibited by adapines (27–34%) and the values seen in the relatively elongate notharctines (38–45%), asiadapines (39–44%), and NMMP 58 (48%, Beard et al. (2007)); *Anchomomys* is unique among stem strepsirrhines in having even longer distal segments (51–53%). The value for NMB Eh 719 matches that which was reported for *Europolemur* by Gebo et al. (2001). The variable “ResB” of Boyer et al. (2013), which is the residual from a regression of absolute distal calcaneal segment length on estimated body mass across primates, is −0.16. Among Paleogene primates, this residual is higher than those exhibited by asiadapines and adapines, but lower than those of notharctines, *Anchomomys*, and all omomyiforms. *Caenopithecus*’ value is lower than those of all extant lemuriforms and galagids, but higher than those of lorisids (see Table 1 of Boyer et al., 2013). Among subfossil lemurs, *Mesopropithecus* has a much higher “Res B” residual, *Megaladapis* and *Pachylemur* have slightly higher residuals, while *Archaeolemur*, *Babakotia*, and *Palaeopropithecus* have much lower residuals.

**Figure 12:**
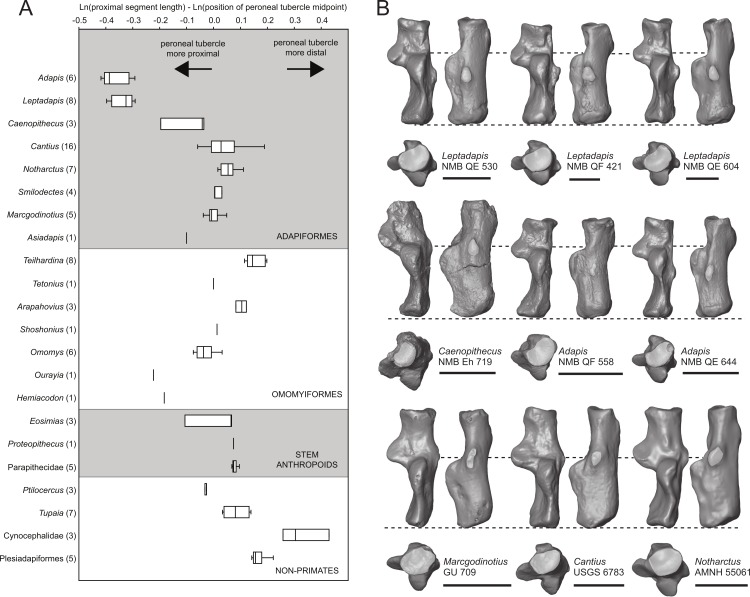
Peroneal tubercle position in living and extinct primates, and comparisons of the *Caenopithecus* calcaneus NMB Eh 719 with those of other adapiforms. (A) Box and whisker plots of ((natural log of proximal segment length)—(natural log of the position of peroneal tubercle midpoint)) measured on the calcanei of 50 Eocene adapiforms, 21 Eocene omomyiforms, four Eocene stem anthropoids, five Oligocene parapithecids, five Paleocene plesiadapiforms, and 15 extant non-primate euarchontans. Note that the range of *Caenopithecus* is intermediate between those of adapines and other adapiforms, but the broad range is largely driven by a single specimen. (B) Calcanei of various adapiforms scaled to the same approximate proximal segment length, illustrating differences in peroneal tubercle position (peroneal tubercles are delimited by opaque overlays). Also note the very short distal calcaneal segments of *Adapis* and *Leptadapis* relative to those of *Caenopithecus* and other adapiforms, and the different shapes and orientations of the long axes of the cuboid facets in distal view (margins of the cuboid facets are also delimited by opaque overlays). Scale bar = 10 mm.

The position of the peroneal tubercle in *Caenopithecus* is also intermediate between the relative placements in adapines and other adapiforms, although *Asiadapis* shows a similar pattern (Fig. 12A and Table 5). *Europolemur klatti* (GMH XXXII-196) has a somewhat proximally-placed peroneal tubercle—clearly proximal to the distal terminus of the ectal facet, and therefore more adapine-like than *Caenopithecus*. NMMP 20 and NMMP 58 have also been interpreted as having adapine-like positions of the peroneal tubercle (Ciochon et al., 2001; Beard et al., 2007). The peroneal tubercles of adapines are particularly well-developed and protrude markedly directly plantar to the apex of the ectal facet; they are so different from those of other adapiforms in their robust construction that Decker & Szalay (1974) even questioned their homology with those of their relatives. In strong contrast, the peroneal tubercles of *Caenopithecus* have proximal and distal borders that grade gradually into the body of the calcaneus.

**Table 5:** Measurements of peroneal tubercle position and size taken on calcanei of living and extinct euarchontans.

Higher taxon	Genus/species	Specimen	(1) ProxL	(2) DistPT	(3) MidPT	(4) (3–1)	(5) (2–1)	(6) [(2–3)–1]
Adapinae	*Adapis parisiensis*	NMB QE 530	14.74	11.82	9.95	−0.393	−0.221	−2.065
Adapinae	*Adapis parisiensis*	NMB QE 644	12.33	9.62	8.22	−0.405	−0.248	−2.176
Adapinae	*Adapis parisiensis*	NMB QE 741	11.95	10.25	7.87	−0.418	−0.153	−1.614
Adapinae	*Adapis parisiensis*	NMB QE 779	13.3	12.05	9.72	−0.314	−0.099	−1.742
Adapinae	*Adapis parisiensis*	NMB QF 558	10.88	8.02	7.39	−0.387	−0.305	−2.849
Adapinae	*Adapis parisiensis*	NMB QH 640	12.81	10.55	9.57	−0.292	−0.194	−2.570
Adapinae	*Leptadapis magnus*	NMB QW 1676	22.56	19.7	16.55	−0.310	−0.136	−1.969
Adapinae	*Leptadapis magnus*	PQ 1746	23.49	19.58	15.8	−0.397	−0.182	−1.827
Adapinae	*Leptadapis magnus*	ACQ 266	24.74	22.05	16.95	−0.378	−0.115	−1.579
Adapinae	*Leptadapis magnus*	ACQ 267	24.63	22.09	17.97	−0.315	−0.109	−1.788
Adapinae	*Leptadapis magnus*	NMB QE 920	24.56	19.94	17.4	−0.345	−0.208	−2.269
Adapinae	*Leptadapis magnus*	NMB QE 604	21.13	17.9	15.61	−0.303	−0.166	−2.222
Adapinae	*Leptadapis magnus*	NMB QF 421	23.15	19.02	16.73	−0.325	−0.197	−2.313
Adapinae	*Leptadapis magnus*	NMB QE 830	21.61	18.6	16.15	−0.291	−0.150	−2.177
Asiadapinae	*Asiadapis cambayensis*	GU 716	5.76	5.89	na	na	0.022	na
Asiadapinae	*Asiadapis cambayensis*	GU 760	6.64	6.71	6.01	−0.100	0.010	−2.250
Asiadapinae	*Marcgodinotius indicus*	GU 1644	4.19	4.98	4.13	−0.014	0.173	−1.595
Asiadapinae	*Marcgodinotius indicus*	GU 709	4.55	5.33	4.52	−0.007	0.158	−1.726
Asiadapinae	*Marcgodinotius indicus*	GU 751	4.82	5.71	5.06	0.049	0.169	−2.004
Asiadapinae	*Marcgodinotius indicus*	GU 1642	4.59	4.9	4.42	−0.038	0.065	−2.258
Asiadapinae	*Marcgodinotius indicus*	GU 1643	4.33	5.21	4.39	0.014	0.185	−1.664
Caenopithecinae	*Caenopithecus lemuroides*	NMB Eh 719	13.12	14.39	12.58	−0.042	0.092	−1.981
Caenopithecinae	*Caenopithecus lemuroides*	NMB En.268	14.12	14.46	11.61	−0.196	0.024	−1.600
Caenopithecinae	*Caenopithecus lemuroides*	NMB En.269	13.76	15.31	13.27	−0.036	0.107	−1.909
Carpolestidae	*Carpolestes simpsoni*	UM 101963	4.12	6.85	5.89	0.357	0.508	−1.457
Cynocephalidae	*Cynocephalus volans*	UNSM 11501	7.92	12.37	10.25	0.258	0.446	−1.318
Cynocephalidae	*Cynocephalus volans*	AMNH 207001	9.08	14.37	12.3	0.304	0.459	−1.479
Cynocephalidae	*Galeopterus variegatus*	USNM 317118	7.5	13.25	11.52	0.429	0.569	−1.467
Eosimiidae	*Eosimias* sp.	IVPP 11851	3.59	4.51	3.84	0.067	0.228	−1.679
Eosimiidae	*Eosimias* sp.	IVPP 12313	4.33	5.98	4.62	0.065	0.323	−1.158
Eosimiidae	*Eosimias* sp.	IVPP 12281	4.08	4.73	3.67	−0.106	0.148	−1.348
Notharctinae	*Cantius mckennai*	USGS 5897	10.45	12.72	11.4	0.087	0.197	−2.069
Notharctinae	*Cantius* sp.	USGS 6791	11.76	12.89	11.71	−0.004	0.092	−2.299
Notharctinae	*Cantius* sp.	USGS 21768	13.96	16.13	14.2	0.017	0.144	−1.979
Notharctinae	*Cantius abditus*	USGS 21771	14.04	16.91	15.23	0.081	0.186	−2.123
Notharctinae	*Cantius abditus*	USGS 21774	13.52	15.12	13.41	−0.008	0.112	−2.068
Notharctinae	*Cantius* sp.	USGS 21778	12.24	13.98	12.71	0.038	0.133	−2.266
Notharctinae	*Cantius abditus*	USGS 21825	13.53	15.06	13.66	0.010	0.107	−2.268
Notharctinae	*Cantius abditus*	USGS 21827	14.25	16.34	13.99	−0.018	0.137	−1.802
Notharctinae	*Cantius frugivorus*	USGS 21828	11.28	12.69	11.11	−0.015	0.118	−1.966
Notharctinae	*Cantius mckennai*	USGS 25029a	11.28	13.25	12.04	0.065	0.161	−2.232
Notharctinae	*Cantius mckennai*	USGS 25029b	11.28	13.1	11.73	0.039	0.150	−2.108
Notharctinae	*Cantius abditus*	AMNH 16852	13.05	15.09	13.75	0.052	0.145	−2.276
Notharctinae	*Cantius abditus*	USGS 6783	13.67	15.95	14.06	0.028	0.154	−1.979
Notharctinae	*Cantius ralstoni*	UF 252980	8.63	11.12	10.43	0.189	0.254	−2.526
Notharctinae	*Cantius* sp.	USGS 21829	12.41	14.61	13.41	0.077	0.163	−2.336
Notharctinae	*Cantius trigonodus*	USGS 6774	14.98	15.21	14.12	−0.059	0.015	−2.621
Notharctinae	*Notharctus tenebrosus*	AMNH 13766	15.46	18.42	16.35	0.056	0.175	−2.011
Notharctinae	*Notharctus tenebrosus*	AMNH 55061	13.27	16.17	13.65	0.028	0.198	−1.661
Notharctinae	*Notharctus tenebrosus*	AMNH 129382	13.56	16.07	14.28	0.052	0.170	−2.025
Notharctinae	*Notharctus tenebrosus*	AMNH 11474	13.25	15.01	13.46	0.016	0.125	−2.146
Notharctinae	*Notharctus tenebrosus*	AMNH 131945	14.26	17.66	15.05	0.054	0.214	−1.698
Notharctinae	*Notharctus tenebrosus*	AMNH 131955	14.79	18.07	16.53	0.111	0.200	−2.262
Notharctinae	*Notharctus tenebrosus*	AMNH 11478	15.57	18.57	16.73	0.072	0.176	−2.136
Notharctinae	*Smilodectes gracilis*	USNM 21815	14.5	16.85	14.95	0.031	0.150	−2.032
Notharctinae	*Smilodectes gracilis*	USNM 25686	14.76	16.38	14.82	0.004	0.104	−2.247
Notharctinae	*Smilodectes gracilis*	AMNH 131763	15.02	17.46	15.45	0.028	0.151	−2.011
Notharctinae	*Smilodectes gracilis*	AMNH 131774	14.85	16.96	14.93	0.005	0.133	−1.990
Omomyiformes	*Arapahovius gazini*	UCMP 118498	3.59	4.33	3.9	0.083	0.187	−2.122
Omomyiformes	*Arapahovius gazini*	UCMP 118499	3.39	4.43	3.83	0.122	0.268	−1.732
Omomyiformes	*Arapahovius gazini*	UCMP 173038	3.79	4.76	4.21	0.105	0.228	−1.930
Omomyiformes	*Hemiacodon gracilis*	AMNH 12613	7.61	8.01	6.34	−0.183	0.051	−1.517
Omomyiformes	*Omomys carteri*	UCM 67678	6.93	7.79	6.52	−0.061	0.117	−1.697
Omomyiformes	*Omomys carteri*	UCM 68745	7.23	8.61	6.97	−0.037	0.175	−1.484
Omomyiformes	*Omomys carteri*	UCM 69065	7.52	9.69	7.13	−0.053	0.254	−1.078
Omomyiformes	*Omomys carteri*	UCM 67679	7.04	8.82	6.53	−0.075	0.225	−1.123
Omomyiformes	*Omomys carteri*	UCM 69303	7.88	9.08	8.14	0.032	0.142	−2.126
Omomyiformes	*Omomys carteri*	UM 98604	6.45	7.01	6.4	−0.008	0.083	−2.358
Omomyiformes	*Ourayia uintensis*	SDNM 4020-60933	10.96	12.58	8.77	−0.223	0.138	−1.057
Omomyiformes	*Shoshonius cooperi*	CM 69765	4.43	5.3	4.49	0.013	0.179	−1.699
Omomyiformes	*Teilhardina belgica*	IRSNB M1236	3.49	4.99	4.03	0.144	0.358	−1.291
Omomyiformes	*Teilhardina belgica*	IRSNB M1237	3.09	4.2	3.63	0.161	0.307	−1.690
Omomyiformes	*Teilhardina belgica*	IRSNB M1247	3.2	4.47	3.9	0.198	0.334	−1.725
Omomyiformes	*Teilhardina belgica*	IRSNB 16786-06	3.27	4.28	3.67	0.115	0.269	−1.679
Omomyiformes	*Teilhardina belgica*	IRSNB 26857-05	3.19	4.12	3.62	0.126	0.256	−1.853
Omomyiformes	*Teilhardina belgica*	IRSNB 26857-04	3.48	4.77	4.15	0.176	0.315	−1.725
Omomyiformes	*Teilhardina belgica*	IRSNB M0061	3.13	4.35	3.62	0.145	0.329	−1.456
Omomyiformes	*Teilhardina belgica*	IRSNB 16786-03	3.31	4.63	4.01	0.192	0.336	−1.675
Omomyiformes	*Tetonius* sp.	AMNH 88821	4.49	6.7	4.49	0.000	0.400	−0.709
Parapithecidae	*Apidium phiomense*	DPC 1003B	14.12	17.39	15.22	0.075	0.208	−1.873
Parapithecidae	*Apidium phiomense?*	DPC 8810	13.45	17.52	14.81	0.096	0.264	−1.602
Parapithecidae	*Apidium phiomense*	DPC 2381	13.79	17.41	15	0.084	0.233	−1.744
Parapithecidae	*Apidium phiomense*	DPC 15679	14.06	17.4	15.05	0.068	0.213	−1.789
Parapithecidae	*Apidium phiomense*	DPC 20576	9.72	12.4	10.45	0.072	0.244	−1.606
Paromomyidae	*Ignacius graybullianus*	USNM 442240	4.82	7.04	6.02	0.222	0.379	−1.553
Plesiadapidae	*Nannodectes gidleyi*	AMNH 17379	6.49	8.95	7.48	0.142	0.321	−1.485
Plesiadapidae	*Plesiadapis churchilli*	UM no number	7.71	10.53	9.01	0.156	0.312	−1.624
Plesiadapidae	*Plesiadapis cookei*	UM 87990	11.13	16.05	12.91	0.148	0.366	−1.265
Plesiadapidae	*Plesiadapis tricuspidens*	MNHN R 414	12.25	17.23	14.65	0.179	0.341	−1.558
Proteopithecidae	*Proteopithecus sylviae*	DPC 24776	7.19	8.45	7.74	0.074	0.161	−2.315
Ptilocercidae	*Ptilocercus lowii*	USNM 488067	3.13	3.74	3.05	−0.026	0.178	−1.512
Ptilocercidae	*Ptilocercus lowii*	USNM 488069	3.13	3.73	3.03	−0.032	0.175	−1.498
Ptilocercidae	*Ptilocercus lowii*	USNM 488072	3.13	3.66	3.05	−0.026	0.156	−1.635
Tupaiidae	*Tupaia belangeri*	AMNH 113135	5.3	7.16	5.51	0.039	0.301	−1.167
Tupaiidae	*Tupaia glis*	SBU Tg01	5.09	7.72	5.26	0.033	0.417	−0.727
Tupaiidae	*Tupaia glis*	AMNH 215175	4.9	7.58	5.63	0.139	0.436	−0.921
Tupaiidae	*Tupaia glis*	AMNH 215176	4.62	6.96	4.91	0.061	0.410	−0.813
Tupaiidae	*Tupaia glis*	AMNH 215177	5.23	7.9	5.92	0.124	0.412	−0.971
Tupaiidae	*Tupaia glis*	AMNH 215178	5.18	7.63	5.91	0.132	0.387	−1.102
Tupaiidae	*Tupaia glis*	AMNH 215179	4.99	6.93	5.41	0.081	0.328	−1.189

**Notes.**

ProxL length of the proximal segment DistPT distance from the proximal-most aspect of the calcaneal tuber to the distal-most projection of the peroneal tubercle MidPT distance from the proximal-most aspect of the calcaneal tuber to the midpoint of the peroneal tubercle (3-1) MidPT—ProxL (2-1) DistPT—ProxL (2-3)—1 [(DistPT-MidPT)—ProxL]

The width/length ratio of the ectal facet of NMB Eh 719 (55) is more similar to those of adapines than notharctines (means of which, reported by Gebo et al. (2001), range from 45–52, but with ranges extending to 60 in *Notharctus pugnax*). The value of 64 reported for NMMP 58 would be particularly high for an adapiform, with similar values only seen in *Adapis*. Based on the values reported by Thalmann (1994), the index for *Europolemur klatti* is particularly low (41.8), at the low end of the notharctine range.

**Figure 13:**
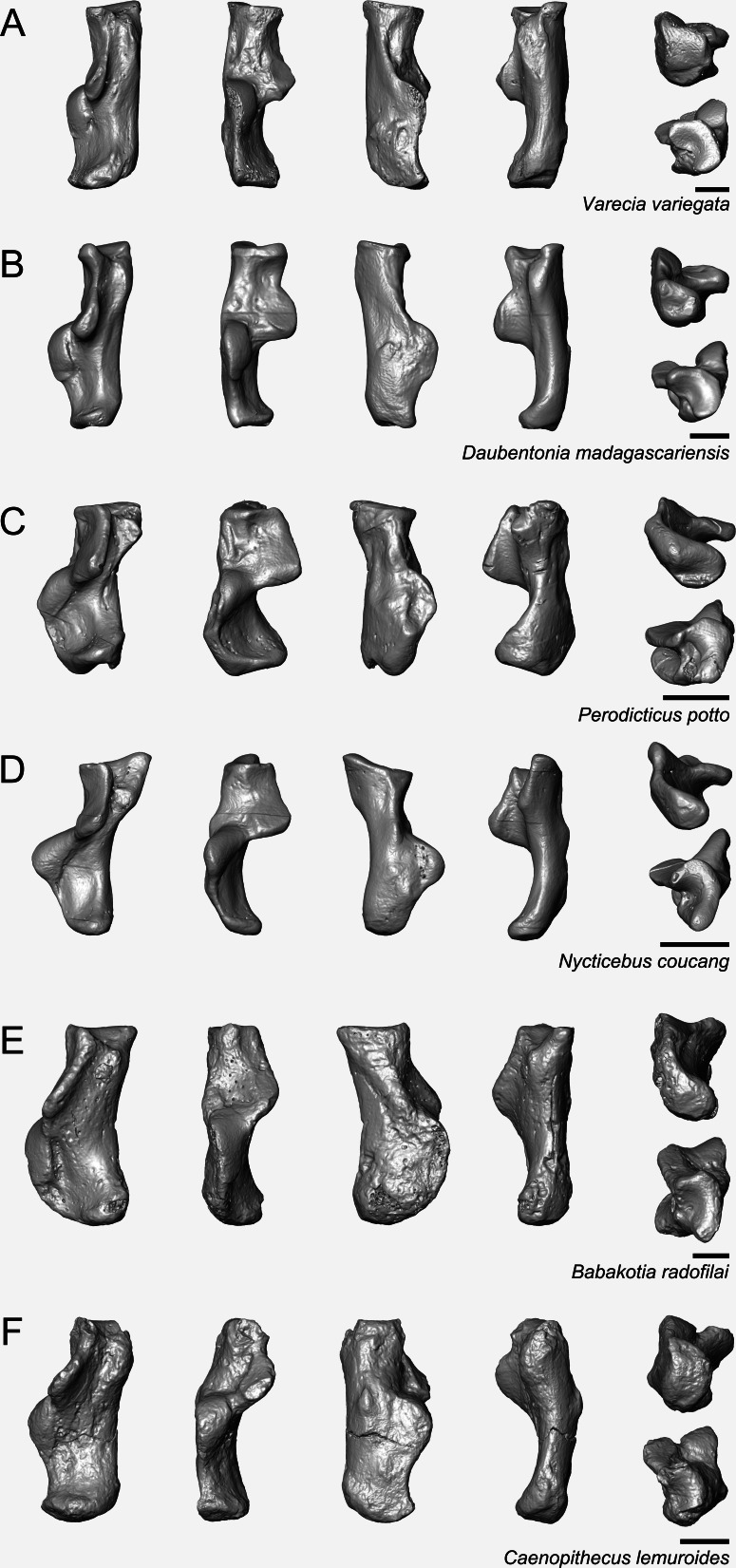
Calcanei of extant and subfossil strepsirrhines compared to NMB Eh 719, attributed here to *Caenopithecus lemuroides*. (A) *Varecia variegata* (AMNH 201384); (B) *Daubentonia madagascariensis* (AMNH 185643); (C) *Perodicticus potto* (AMNH 184597); (D) *Nycticebus coucang* (AMNH 102027); (E) *Babakotia radofilai* (DPC 11818); (F) *Caenopithecus lemuroides* (NMB Eh 719). Views in, from left to right, medial, dorsal, lateral, and plantar; and, on the far right, proximal (above) and distal (below). Scale bars are equal to 5 mm.

The cuboid facets of *Caenopithecus* are similar to those of adapines in being fan-shaped, but the long axes of the facets in the former are more dorsoplantarly oriented than the latter (Fig. 12). A similarly dorsoplantar orientation of the cuboid facet appears to be present in NMMP 20 (Ciochon et al., 2001) and NMMP 58 (Beard et al., 2007), and, among crown strepsirrhines, in lorisids and *Babakotia* (Fig. 13). Notharctines, asiadapines, and *Anchomomys* have cuboid facets whose long axes are oriented more mediolaterally than dorsoplantarly.

The well-developed distal calcaneal tubercles of *Caenopithecus* (particularly notable in NMB Eh 719 and NMB En.269) are similar to those of adapines, which typically (but not consistently) have better-developed tubercles than notharctines such as *Cantius* and *Notharctus*. *Anchomomys*, *Marcgodinotius*, and NMMP 58 also have relatively small distal calcaneal tubercles.

The dorsolateral inclination of the sustentacular facet in *Caenopithecus* is—when judged relative to the mediolateral plane of the ectal facet—also seen in basal adapiforms such as *Cantius* and *Marcgodinotius*; other adapiforms, including adapines and more basal taxa such as *Asiadapis*, show a larger angle between the mediolateral planes of the ectal and sustentacular facets.

The strong medial bowing of the calcaneal tuber in *Caenopithecus*, combined with its lack of development of any lateral flaring, appears to be unique among adapiforms, but among extant primates is seen in lorisids (Fig. 13). The preserved portion of the calcaneal tuber of NMMP 58 appears to be bowed medially, but the most proximal portion of the tuber is damaged.

### Automated geometric analysis of primate calcanei

**Figure 14:**
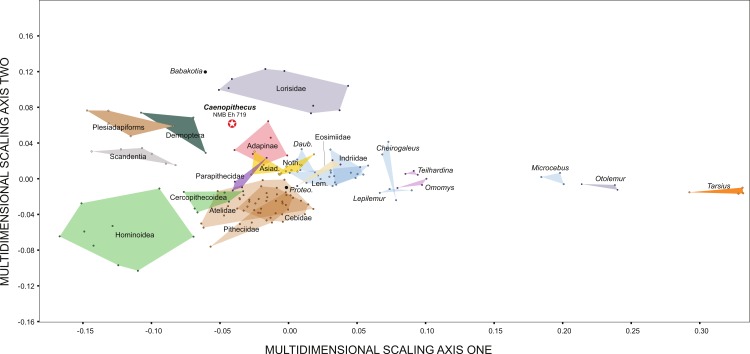
Multidimensional scaling plot derived from automated geometric morphometric analysis of euarchontan calcanei, based on 1,200 points. The position of *Caenopithecus lemuroides* (based on NMB Eh 719) is marked by a white star enclosed in a red circle. Abbreviations: “Asiad,” Asiadapinae; “*Daub*,” *Daubentonia*; “Noth,” Notharctinae; “*Proteo*,” *Proteopithecus*.

The multidimensional scaling (MDS) plot from the *auto3dgm* analysis (Fig. 14; see Datasets S3 and S4 for 3D coordinates and MDS coordinates, respectively) appears to capture a mix of phylogenetic and functional signals. The first axis is probably related in large part to major differences in distal calcaneal elongation, with the relatively elongate calcanei of tarsiers, galagids, *Microcebus*, and omomyiforms having strongly positive scores, and the relatively foreshortened calcanei of non-primate euarchontans, hominoids, and cercopithecoids having strongly negative scores. Several basal extinct primate clades, such as Notharctinae, Adapinae, Asiadapinae, and Eosimiidae occupy a central zone along the first axis, presumably reflecting in part their intermediate levels of distal calcaneal elongation. *Caenopithecus* (NMB Eh 719) falls into a unique part of the primate calcaneal morphospace, showing no overlap with any other living or extinct taxon, but falling about mid-way between the polygons encompassing Dermoptera, Lorisidae, and Adapinae; *Babakotia* also falls into this part of the MDS plot, but is in an even more extreme position, actually falling outside of the morphospace encompassing all other known living and extinct primates. Non-cheirogaleid, non-lepilemurid lemuriforms show considerable cohesion along the first axis despite variation in locomotor style, presumably reflecting strong phylogenetic signal in calcaneal morphology; the same can be said of platyrrhines as a whole. Lorisids fall within the lemuriform morphospace along the first axis, but, along with *Babakotia*, some adapines, and *Caenopithecus*, have strongly positive scores along the second axis. Overall, as with the multivariate analysis of the astragalus, *Caenopithecus* appears to occupy a calcaneal morphospace that is best interpreted as being related to cautious slow climbing, considerable pedal mobility, and possibly hind limb suspension. The proximity of *Caenopithecus* to adapines is consistent with its phylogenetic placement (see below), while its more positive score along the second axis (relative to most adapines) might reflect an increased emphasis on slow and cautious climbing, and possibly hind limb suspension, when compared to its adapine relatives. Such a scenario is also consistent with the functional interpretation that has been put forth for the closely related caenopithecine *Afradapis*, and the possible caenopithecine *Adapoides*, on the basis of their astragalar morphology, which is very loris-like (Boyer, Seiffert & Simons, 2010). We further explore the functional implications of *Caenopithecus*’ tarsal morphology below.

### Phylogenetic analysis

**Figure 15:**
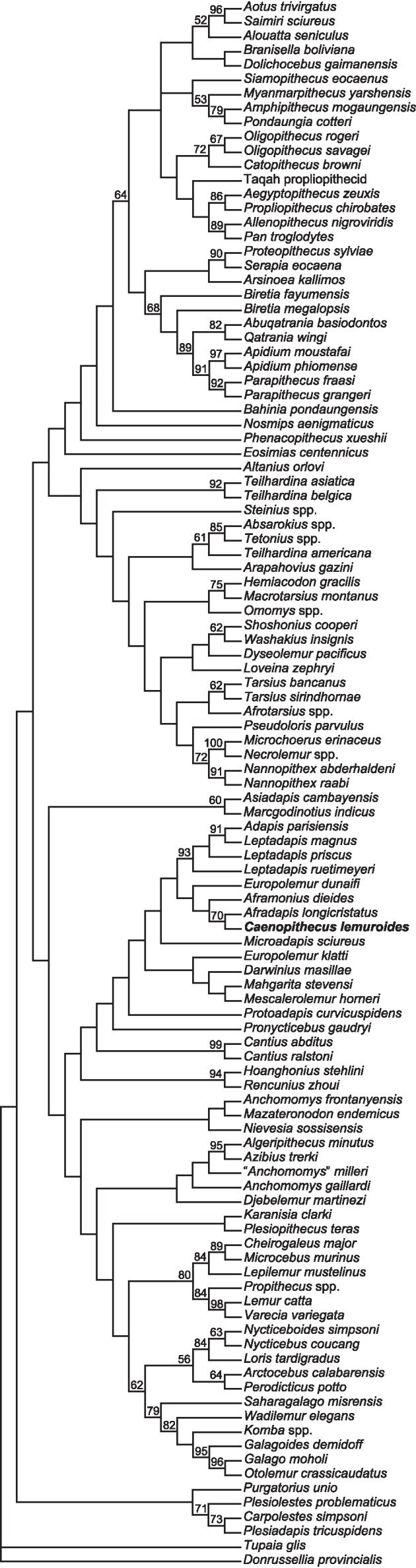
Phylogenetic analysis with some multistate characters ordered. Strict consensus of two equally parsimonious trees of length 4330.5 recovered following parsimony analysis (10,000 heuristic search replicates) of the 291 character matrix in PAUP* 4.10b, with 256 of the characters ordered and transitions between “fixed” and “polymorphic” states in ordered morphoclines weighted as 0.5. See text for tree statistics; numbers above or below branches are bootstrap values, following 1,000 pseudoreplicates. Relationships among extant species were constrained by a molecular “scaffold” following Springer et al. (2012).

Parsimony analysis of the character matrix with 256 of 291 characters ordered, transitions between fixed and polymorphic states weighted as a half-step, premolar re-acquisition not allowed, and with the molecular scaffold enforced recovered two trees of 4330.5 steps (consistency index excluding uninformative characters (CI) = 0.1605, retention index (RI) = 0.5646, rescaled consistency index (RC) = 0.0914, Fig. 15). The only differences between the current result and that of Boyer et al. (2015b, who provided the most recent modification of the matrix used here) are (1) the placement of the clade (*Europolemur dunaifi*, (*Aframonius*, (*Afradapis*, *Caenopithecus*))) as the sister taxon of Adapinae to the exclusion of *Microadapis* (and all other adapiforms), (2) the placement of taxa previously situated as basal caenopithecines (*Europolemur klatti* and *Mahgarita stevensi*) as members of a (*Europolemur klatti*, (*Darwinius*, (*Mahgarita*, *Mescalerolemur*))) clade, and (3) notharctines (represented by *Cantius*) and the “cercamoniines” *Pronycticebus* and *Protoadapis* are consecutive sister taxa of a clade containing all of the aforementioned taxa. None of these relationships are well-supported—only the Adapinae (bootstrap value of 93), *Adapis parisiensis* + *Leptadapis magnus* (91), and *Afradapis* + *Caenopithecus* (70) clades are supported by bootstrap values higher than 50.

**Figure 16:**
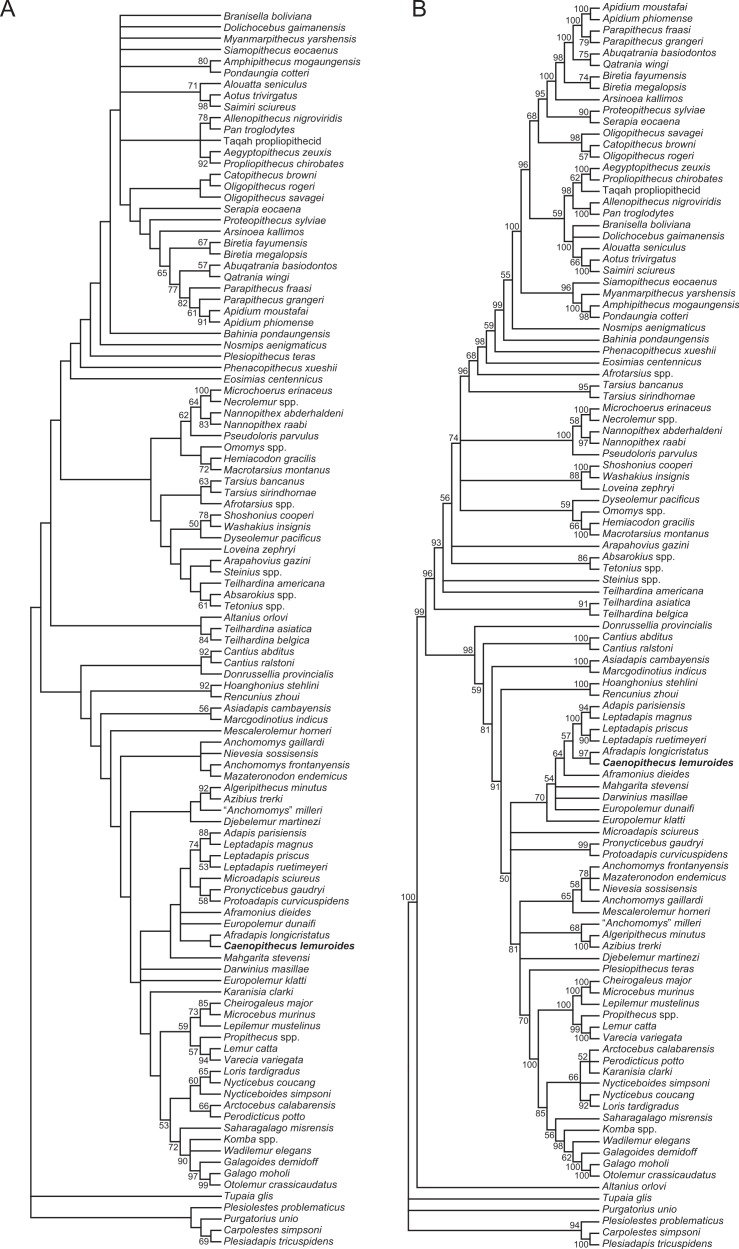
Phylogenetic analysis with all characters unordered. (A) Strict consensus of 342 equally parsimonious trees of length 4,638 following parsimony analysis (10,000 heuristic search replicates) of the 391 character matrix in PAUP* 4.10b, with all characters unordered and equally weighted. See text for tree statistics; numbers above or below branches are bootstrap values, following 1,000 pseudoreplicates. Relationships among extant species were constrained by a molecular “scaffold” following Springer et al. (2012). Note that, unlike the consensus tree with some characters ordered and scaled, adapines form a clade with *Microadapis*, *Pronycticebus*, and *Protoadapis* rather than with any caenopithecine, though *Caenopithecus* still forms a clade with *Afradapis* to the exclusion of all other species. (B) “Halfcompat” (majority-rule) consensus tree following 50 million MCMC generations in MrBayes (first 25% discarded as “burn-in”). Numbers above or below branches are posterior probabilities. Relationships among extant species were constrained by a molecular “scaffold” following Springer et al. (2012). Note that caenopithecines are paraphyletic with respect to adapines given this topology, unambiguously implying re-acquisition of the upper and lower first premolar in the latter clade.

Parsimony analysis with all characters unordered and equally weighted, but constrained by the molecular scaffold, led to the recovery of 342 equally parsimonious trees of length 4,638 (CI = 0.2218, RI = 0.4950, RCI = 0.1104) (Fig. 16A). In contrast to the results from the analysis with some characters ordered and scaled, the sister taxon of Adapinae was found to be a (*Microadapis*, (*Pronycticebus* + *Protoadapis*)) clade rather than Caenopithecinae; an *Afradapis*-*Caenopithecus* clade was placed as a more basal sister group of that clade, alongside *Aframonius* and *E. dunaifi*, followed more distantly by *Magharita*. *Darwinius*, *Djebelemur*, *E. klatti*, *Mescalerolemur*, a European anchomomyin clade, and an African “*Anchomomys*” *milleri* + Azibiidae clade were all placed outside of this clade, but higher up the strepsirrhine stem lineage than notharctines, asiadapines, and sivaladapids.

Both Bayesian phylogenetic analyses presented here reached convergence, judging from very low (<0.01) average standard deviations of split frequencies in the last generations sampled. The “halfcompat” consensus derived from Bayesian analysis with all characters unordered (Fig. 16B) provides more resolution than the comparable parsimony analysis, but few higher-level relationships among adapiforms are well-supported aside from Adapinae (posterior probability (PP = 100)), *Afradapis* + *Caenopithecus* (97), *Pronycticebus* + *Protoadapis* (99), Sivaladapidae (100), and Asiadapinae (100). This analysis differs from both parsimony analyses in placing an *Afradapis*-*Caenopithecus* clade as the sister group of Adapinae to the exclusion of *Aframonius*, though with very weak support. *Darwinius*, *E. dunaifi*, and *Mahgarita* are placed in an unresolved polytomy outside of that clade, followed more distantly by *E. klatti*. One of the more notable implications of this topology is that, given parsimony optimization, it unequivocally requires re-acquisition of the upper and lower first premolar, and re-evolution of a double-rooted upper and lower second premolar (i.e., from a single-rooted condition), along the stem leading to Adapinae. Also notable in this context is the placement of the possible caenopithecine *Mescalerolemur* as a sister taxon of Anchomomyini, far from *Mahgarita*. We suspect that this result is incorrect, given that *Mescalerolemur* and *Mahgarita* are such geographically and temporally unique records in the primate fossil record (and share a number of striking morphological apomorphies), but it is nevertheless interesting that the older and arguably more primitive *Mescalerolemur* is placed closer to crown strepsirrhines than caenopithecines, adapines, and non-anchomomyin “cercamoniines”, similar to the results recovered by Kirk & Williams (2011) for *Mescalerolemur* + *Mahgarita*. Outside of Strepsirrhini, is also notable that, within Haplorhini, results are radically different from those in the parsimony analyses in supporting a “strict tarsier-anthropoid clade” (i.e., tarsiers join anthropoids to the exclusion of all omomyiforms) rather than a monophyletic Tarsiiformes (tarsiers + omomyiforms).

**Figure 17:**
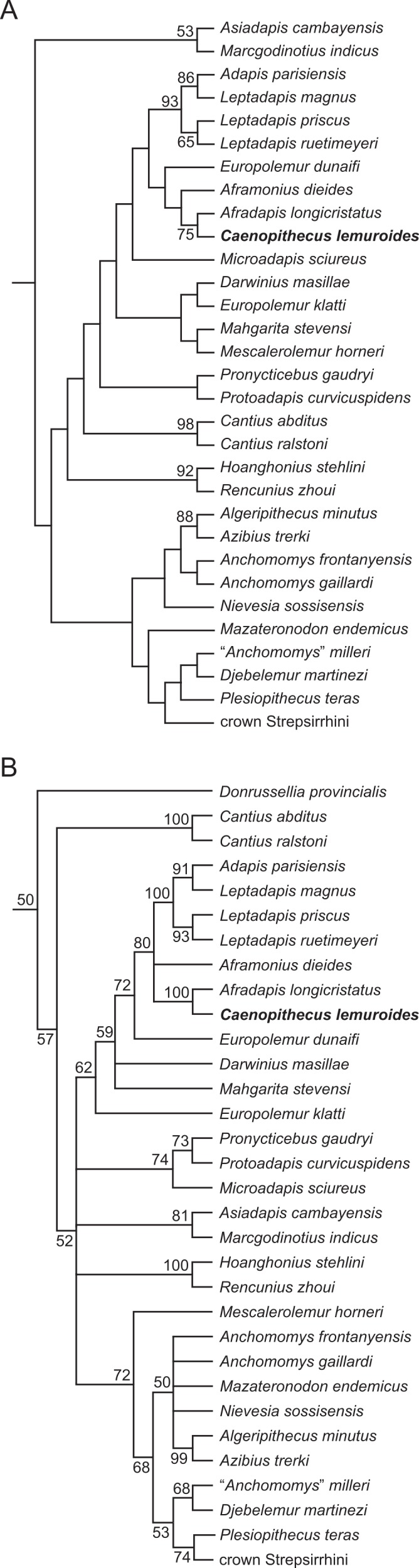
Phylogenetic analysis with standard polymorphic scoring and 256 multistate characters treated as ordered. (A) Strict consensus of 783 equally parsimonious trees of length 3,796 following parsimony analysis (10,000 heuristic search replicates) of the 291 character matrix in PAUP* 4.10b, with all characters equally weighted, “standard” scoring of polymorphisms, and 256 multistate characters treated as ordered. See text for tree statistics; numbers above or below branches are bootstrap values, following 1,000 pseudoreplicates. Relationships among extant species were constrained by a molecular “scaffold” following Springer et al. (2012). (B) “Halfcompat” (majority-rule) consensus tree following 50 million MCMC generations of the same matrix in MrBayes (first 25% discarded as “burn-in”). Numbers above or below branches are posterior probabilities. Relationships among extant species were constrained by a molecular “scaffold” following Springer et al. (2012). Note that, as in the Bayesian analysis of unordered characters caenopithecines are paraphyletic with respect to adapines given this topology, unambiguously implying re-acquisition of the upper and lower first premolar in the latter clade.

Parsimony and Bayesian analysis of the matrix with standard polymorphic scoring (which reduced the total number of states for each of the 256 multistate characters that were ordered in the parsimony analysis, thereby allowing them to be treated as ordered in MrBayes) resulted in some differences from the parsimony results in Figs. 15 and 16A, and the Bayesian results in Fig. 16B, but none that impacted the placement of *Caenopithecus* close to *Afradapis*, and, more distantly, Adapinae (Figs. 17A and 17B). In the parsimony analysis with standard polymorphic scoring (783 equally parsimonious trees of length 3796; CI excluding uninformative characters = 0.1771, RI = 0.5919, RCI = 0.1059), the only changes among stem strepsirrhines were outside of Adapidae, specifically (1) the joining of *Darwinius* and *Europolemur klatti* as a sister clade of *Mahgarita* + *Mescalerolemur* (rather than being paraphyletic with respect to *Mahgarita* + *Mescalerolemur*), (2) recovery of a *Pronycticebus* + *Protoadapis* clade as sister of the clade containing Adapidae, *Darwinius*, *E. klatti*, *Mahgarita*, and *Mescalerolemur* (rather than being paraphyletic with respect to that clade), (3) recovery of a clade containing European *Anchomomys* species and movement of African “*Anchomomys*” *milleri* to a sister taxon relationship with *Djebelemur*, and (4) placement of *Mazateronodon* as the sister taxon of a ((*Plesiopithecus*, (“*A*.” *milleri*, *Djebelemur*)), crown Strepsirrhini) clade.

The Bayesian analysis with 256 characters ordered showed more differences from the results based entirely on unordered characters, but again *Afradapis* was placed as the sister taxon of *Caenopithecus*, and, with *Aframonius*, all caenopithecines were placed as sister taxa of Adapinae, but with higher support (posterior probability of 79 versus 66). There was also moderate support (PP = 72) for placement of *E. dunaifi* as the sister taxon of that clade. There was no support for a clade containing all stem and crown strepsirrhines to the exclusion of asiadapines, notharctines, and *Donrussellia* (supported by a PP of 90 in the analysis with all characters unordered); instead sivaladapids, asiadapines, and a (*Microadapis* (*Pronycticebus*, *Protoadapis*)) clade were placed in a basal polytomy, with only notharctines and *Donrussellia* being placed more basally. The anchomomyin clade broke down into a polytomy with azibiids, and *Mescalerolemur* was again separated from *Mahgarita* and placed as a sister of a clade containing anchomomyins, azibiids, djebelemurids, *Plesiopithecus*, and crown strepsirrhines.

When the same phylogenetic analyses are run with the Egerkingen tarsals scored as *Leptadapis priscus* rather than as *Caenopithecus*, the results of some analyses were different, though not radically so. Under parsimony (with 256 characters ordered, transitions between fixed and polymorphic states weighted as a half-step, premolar re-acquisition not allowed, and with the molecular scaffold enforced) *Caenopithecus* still formed a clade with *Afradapis*, but caenopithecines as a whole were not placed as the closest sister taxa of Adapinae; rather, the sister group of the “core” caenopithecines (i.e., (*Aframonius*, (*Afradapis*, *Caenopithecus*))) was a clade containing *Darwinius* and *E. dunaifi*. *Mescalerolemur* and *Mahgarita* together formed the sister group of that larger clade, and all of those taxa were joined more basally by *E. klatti*, while *Microadapis* was placed as the sister taxon of Adapinae. When all characters were treated as unordered, parsimony analysis placed adapines as the sister taxon of a (*Microadapis*, (*Pronycticebus*, *Protoadapis*)) clade, and caenopithecines (broadly defined) were paraphyletic with respect to that larger clade. Under Bayesian inference, the support for the paraphyly of caenopithecines with respect to adapines disappeared, and there was only very weak support (0.51 posterior probability) for an (*Aframonius*, (*Afradapis*, *Caenopithecus*)) clade, and no support (i.e., posterior probability of <50) for a caenopithecine-adapine clade. With the change to the treatment of polymorphic characters (i.e., use of “standard” polymorphic scoring), the same strict consensus and “halfcompat” topologies were retrieved regardless of whether the Egerkingen tarsals were attributed to *L. priscus* or *Caenopithecus*.

## Discussion and Conclusions

### Functional considerations

In her doctoral dissertation, Dagosto argued that “*Adapis parisiensis*, *Leptadapis magnus*, and *Caenopithecus lemuroides* have features of the upper ankle joint and foot proportions which strongly suggest that quadrupedal slow climbing was the dominant form of locomotion” (Dagosto, 1986, p. 333). Our analyses lend additional support to this hypothesis, and our functional interpretation is broadly consistent with that of Dagosto (1986).

It has been argued that several features of the strepsirrhine hind limb, including astragalar features such as laterally flaring fibular facets and laterally placed grooves for the tendon of flexor fibularis, reflect an ancestral dependence on the use of inverted and abducted foot postures on inclined and vertical small-diameter supports (Gebo, 2011). Some features of the *Caenopithecus* tarsals suggest that, relative to other adapiforms, *C. lemuroides*’ tarsus may have been held in particularly inverted postures. For instance, if the fibular facet angle faithfully reflects pedal inversion (Gebo, 2011; Boyer & Seiffert, 2013), then *Caenopithecus*’ high value alone would suggest that this taxon might have had more inverted foot postures than any other Paleogene primate aside from *Afradapis*, *Cantius nuniensis*, and *Djebelemur* (though the ranges of *Adapis parisiensis* and *Notharctus tenebrosus* also encompass values as high as that of *Caenopithecus*, see Boyer & Seiffert (2013)). Boyer et al. (2015a) and Boyer et al. (2015b) have also recently argued that the ratio of medial tibial facet (MTF) area to ectal facet (EF) area provides another quantitative proxy for pedal inversion in fossil primates, and *Caenopithecus*’ ratio of 0.11 is higher than that of any other adapiform. Among extant primates, *Caenopithecus*’ value is higher than those of cheirogaleids, *Lepilemur*, *Daubentonia*, and even some lorises, but is equaled or exceeded by leaping galagids, indriids, and lemurids. Boyer et al. (2015b) argued that the high MTF/EF area ratios of the latter strepsirrhines might reflect increased loading of the MTF due to acrobatic grasp-leaping, i.e., increased loading relative to more generalized (less acrobatic) ancestors whose feet were already more habitually inverted than those of other, non-strepsirrhine primates, and thus already had high MTF/EF area ratios.

Given that extant strepsirrhine grasp-leapers have both high fibular facet angles and high MTF/EF area ratios as in *Caenopithecus*, it could be argued that the latter’s values reflect acrobatic leaping as well as pedal inversion. However, we consider this to be less likely than a dependence on slow and cautious climbing, and possibly hind limb suspension, when other loris-like features of *Caenopithecus*’ tarsals are taken into account, such as its high astragalar neck angle, inferred presence (based on neck width) of a broad astragalar head, a curved distal projection of the medial tibial facet that extends onto the astragalar neck, and a deeply excavated groove for the tendon of flexor fibularis on the plantar surface of the body (which, at least among extant strepsirrhines, is deepest in species that are known to habitually engage in hind limb suspension). Calcaneal features seen in *Caenopithecus* that are likely related to increased pedal inversion and overall mobility rather than acrobatic grasp-leaping include the dorsoplantarly oriented long axis of the cuboid facet, projection of the ectal facet dorsal to the calcaneal tuber (rather than being level to it), the dorsolateral inclination of the sustentacular shelf, and the convexity of the lateral border of the calcaneal body and (correlated) medial bowing of the calcaneal tuber. Furthermore, the distal calcaneus of *Caenopithecus* is less elongate (relative to body mass) than are the calcanei of acrobatic lemuriforms with high MTF/EF ratios. Finally, in our morphometric analyses the tarsals of *Caenopithecus* show no close phenetic affinities to those of extant leaping primates, and instead appear to be most consistent with a somewhat loris-like complex derived from a generalized adapiform *Bauplan* that combined both notharctine-like and adapine-like features.

**Figure 18:**
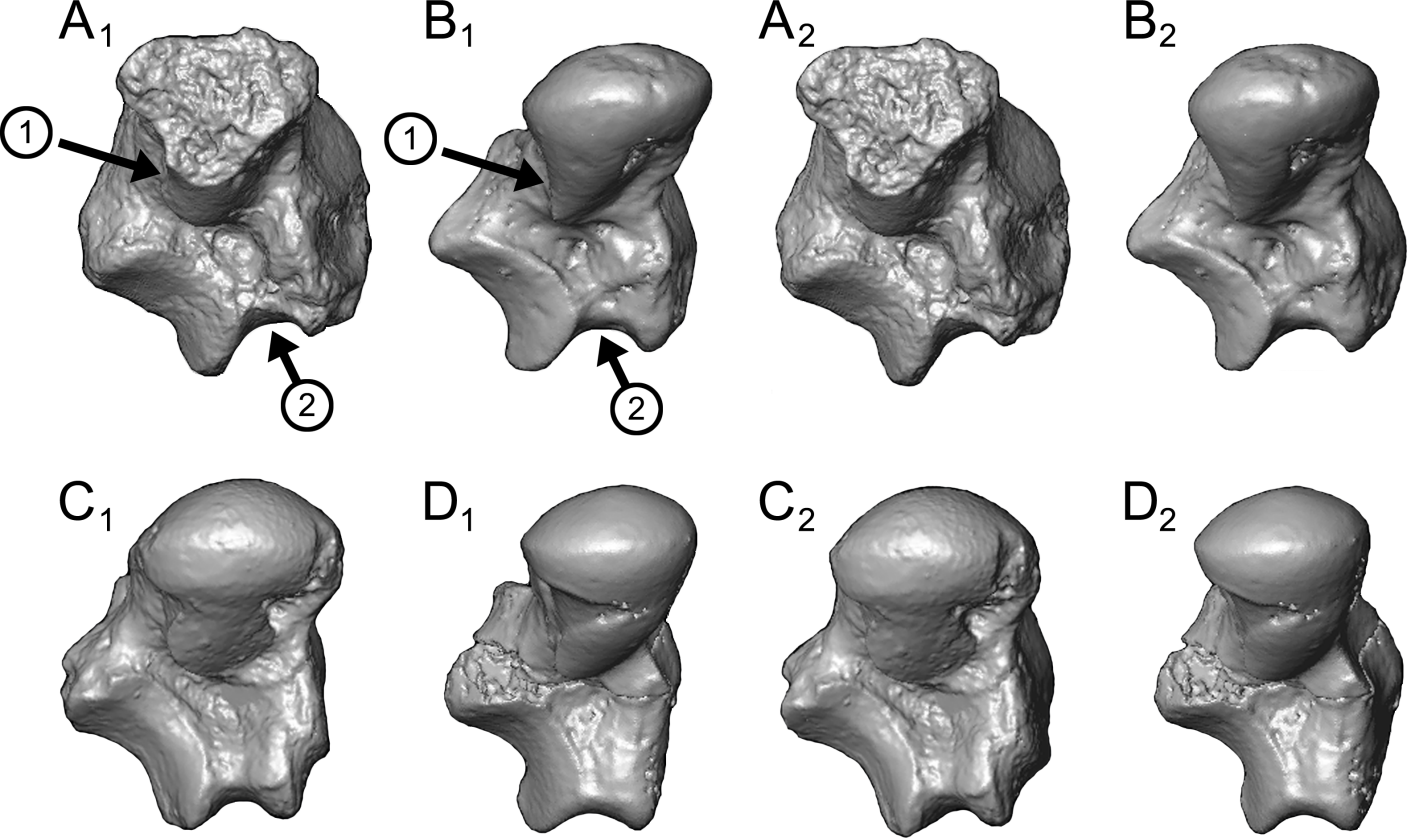
Sustentacular facet morphology and flexor fibularis groove depth in *Caenopithecus* and other adapiforms. Stereopairs of (A) *Caenopithecus* (NMB En.270, reversed); (B) *Afradapis* (DPC 21445C, reversed); (C) *Adapis* (ECA 7377); and *Asiadapis* (GU 747) in distal view, showing (1) the laterally expanded convexity of the sustentacular facet in *Afradapis* and particularly *Caenopithecus*, and (2) the deep flexor fibularis grooves of *Afradapis* and *Caenopithecus* when compared with *Adapis* and *Asiadapis*.

The trochlea-like groove for the tendon of flexor fibularis (the medial wall of which is highly abraded on NMB En.270, but obviously quite prominent, see Fig. 18, feature 2) presumably served to prevent medial slippage of that tendon and maintain its line of action while the foot was held in diverse inverted postures, including those that would have been required by hind limb suspension. The strong medial buttressing of this groove via a bony plantar projection might have been particularly important for *Caenopithecus* because, unlike most other primates, the plantar groove guiding the passage of the flexor fibularis tendon along the plantar surface of the calcaneal sustentaculum was poorly developed, as might be expected given the dorsolateral inclination of the sustentacular shelf. The plantar projection on the medial aspect of the astragalar body might, then, represent a compensatory feature that maintained a tunnel-like passage for the tendon. The continuation of this trochlear passage for the flexor fibularis tendon onto the plantar surface of the astragalus could have been particularly important for the maintenance of strong pedal grasps when the foot was held in extreme plantarflexion (as would occur during hind limb suspension), as this groove and its associated ligaments would have formed—and maintained the integrity of—the so-called “tarsal tunnel” (e.g., Keck, 1962), a structure that, in the case of suspensory species that habitually hold the foot in extreme plantarflexion, might also prevent plantar bowstringing of the flexor fibularis tendon. This inference is supported by the presence of a similarly deep plantar exposure of the flexor fibularis groove in occasionally or habitually suspensory euarchontans such as *Cynocephalus*, subfossil palaeopropithecid “sloth lemurs,” lorises, and even in the occasionally suspensory lemurid *Varecia variegata* (Meldrum, Dagosto & White, 1997), a species whose flexor fibularis grooves are comparatively more shallow than the former taxa, but are nevertheless larger and deeper than those of its close lemurid relatives that rarely, if ever, engage in hind limb suspension (Fig. 11). All of these species also resemble *Caenopithecus* in lacking the distinct posterior trochlear shelf that is seen various Paleogene primates (e.g., notharctine adapiforms and microchoerine omomyiforms) whose postcranial morphology suggests an increased capacity for leaping; the absence of such a shelf-like projection along the proximal border of the lateral tibial facet presumably would have allowed for increased plantarflexion relative to those taxa that have a shelf. A prominent medial wall of the flexor fibularis groove that is similar to that of *Caenopithecus* is also present in *Babakotia* (Fig. 9H). In extant lorises the same function is achieved in a slightly different manner; rather than having a mediolaterally restricted plantar projection, instead the lateral tibial facet (=trochlear articular surface) is mediolaterally broad and its entire surface projects plantar to the flexor fibularis groove (Figs. 9E and 9F), providing a structurally somewhat different (but functionally similar) strong medial buttress for a similarly trochlear groove—again presumably preventing medial slippage and bowstringing of the flexor fibularis tendon, and maintaining the line of action of the tendon during strong pedal grasping and hind limb suspension. Quantification of flexor fibularis groove depth demonstrates that *Afradapis* and *Caenopithecus* are both loris-like in this feature (Fig. 11), while *Babakotia* and *Megaladapis* show the same pattern but take it to an extreme, with values that are much higher than those of any other strepsirrhines. Though *Varecia* isn’t the only lemurid that engages in hind limb suspension (and it should be noted that occasional use of hind limb suspension is not at all uncommon among primates (e.g., Meldrum, Dagosto & White, 1997)), it does so more frequently, and its high values relative to its lemurid relatives might also be explained as a correlate of this behavioral pattern. The high value of *Cheirogaleus medius* is interesting given evidence from that species’ axial skeleton for a more loris-like dorso-stable *Bauplan* than other cheirogaleids (including *Cheirogaleus major*) (Granatosky et al., 2014). The high value of the terrestrial subfossil form *Archaeolemur* is more difficult to explain and requires further investigation, though it does not approach the magnitude seen in *Afradapis* and *Caenopithecus*. In summary, the pronounced height of the astragalar body in *Caenopithecus*—which is often associated with leaping propensities in primates—seems more likely to be a correlate of the plantar projection that buttresses the flexor fibularis groove, while at the same time being accentuated by the absence of the posterior shelf. We interpret these correlated features as two aspects of a morphological pattern that more likely facilitated inversion, increased capacity for plantarflexion, and possibly hind limb suspension in this species.

**Figure 19:**
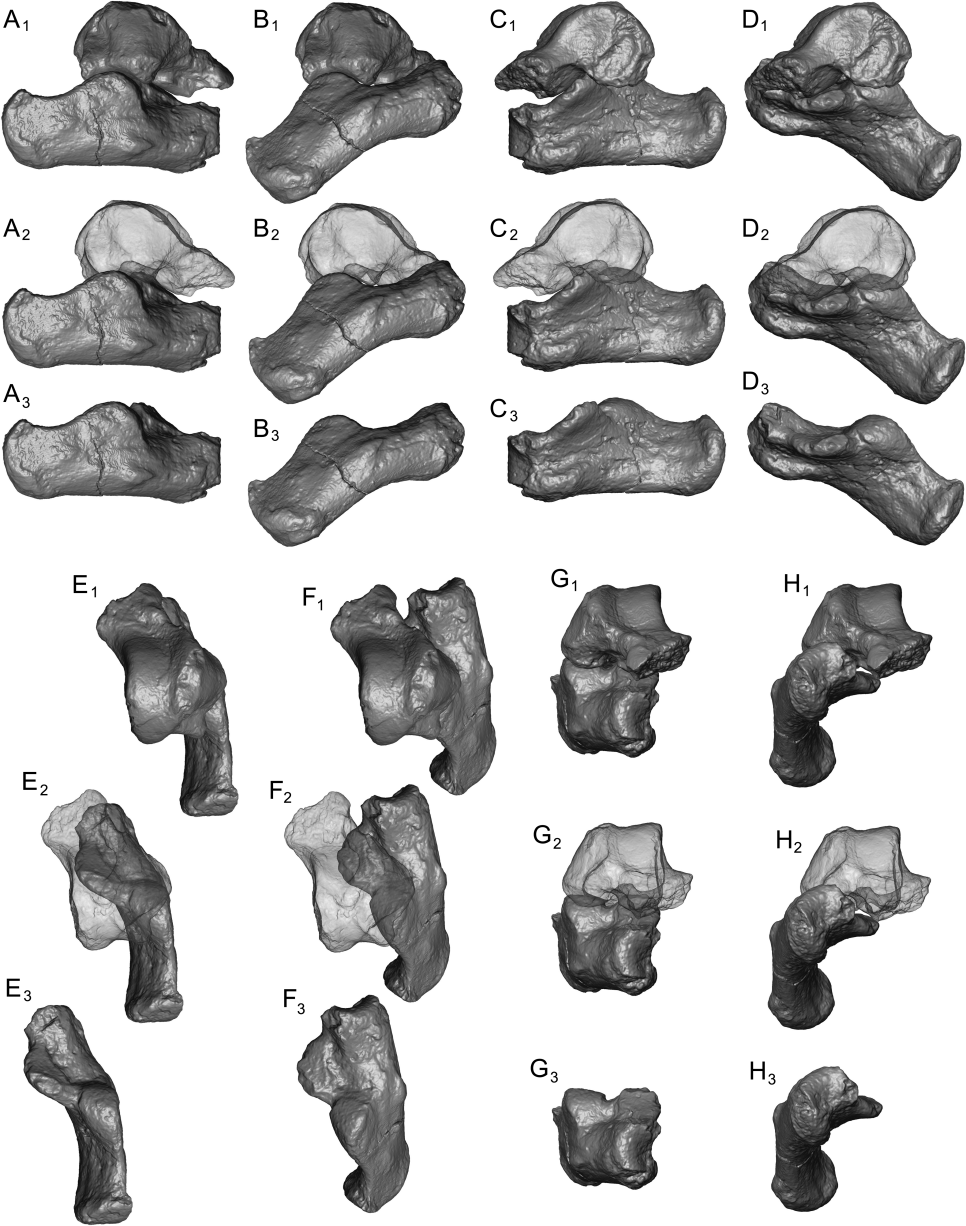
Articulation of the unassociated *Caenopithecus* astragalus NMB En.270 and and calcaneus NMB Eh 719 in inversion and eversion. Articulated astragalus and calcaneus in (A) lateral view, inverted; (B) lateral view, everted; (C) medial view, inverted; (D) medial view, everted; (E) dorsal view, inverted; (F) dorsal view, everted; (G) distal view, inverted; (H) distal view, everted. In each unique view, from top to bottom the astragalus is rendered as solid (e.g., *A*_1_), translucent (e.g., *A*_2_), or is not shown (e.g., *A*_3_).

The dorsoplantar orientation of the long axis of the cuboid facet (and correlated medial placement of the cuboid pivot) is also seen in slow-moving lorises and palaeopropithecids, and suggests that the articulating cuboid would have been held in such a position that the navicular articulation was more dorsally oriented than in those taxa that have cuboid facets whose “pits” are situated along the plantar margin of the facet. The dorsolateral inclination of the calcaneal sustentacular facet might represent a structural mechanism that would have restricted movement of the astragalar neck such that, in the most stable positions allowed by the articulating ectal and sustentacular facets, the long (roughly mediolateral) axis of the astragalar neck and head would have been held in an inverted position. A similar dorsolateral orientation of the sustentaculum, with no plantar groove, is also seen in *Babakotia* (Fig. 13E). The tightly curved calcaneal ectal facet of *Caenopithecus* nevertheless appears to provide an extensive surface for articulation of the astragalar ectal facet, thereby allowing for considerable mobility in the proximodistal plane and corresponding potential for inversion (when positioned relatively proximally) and eversion (when positioned relatively distally) of the calcaneus with respect to the astragalus (Fig. 19, and see Decker & Szalay, 1974) (though this assessment is based on articulation with NMB En.270, which probably does not belong to the same individual, see Fig. 19). This capacity for eversion is interesting given the strikingly convex shape of the proximal portion of the sustentacular facet of *Caenopithecus* (Fig. 16A), which is fairly unique among adapiforms (see the relatively flat sustentacular facets of *Adapis* and *Asiadapis*, Figs. 18C and 18D) and rare among extant primates. Given that the calcaneal sustentacular facet is relatively flat (mediolaterally), the convexity of the articulating astragalar sustentacular facet, and the medioplantar orientation of the medial extension of that facet, suggests a mechanism by which the astragalus might be capable of slipping into a more or less “locked” (but not particularly stable) position when the calcaneus is everted relative to the astragalus (Figs. 19B, 19D, 19E and 19H). Such everted positions greatly reduce the overlap of the articulating sustentacular facets, however, and furthermore lead to a corresponding separation of the head of the astragalus from the distal calcaneus (and, presumably, correlated separation of the navicular from the cuboid), suggesting to us that these foot postures are unlikely to have been used habitually. It is conceivable that such postures might have been employed during quadrumanous bridging behaviors, for instance when one foot maintains a strong grasp on an inclined support, but becomes increasingly everted as the body turns away from that support to grasp a nearby branch with the other limbs. A similar medioplantar extension of the astragalar sustentacular facet is also seen in lorisids, lending some support to that interpretation, but in these taxa the facets are not nearly as convex as that of *Caenopithecus*.

Among living primates, projection of the ectal facet dorsal to the calcaneal tuber is seen in generally slow-moving species such as lorisids, whereas leaping species that depend on powerful propulsion via the triceps surae musculature (such as tarsiers; Gebo, 1987), tend to have calcaneal tubers that project dorsally to approximately the same level as the most dorsal surface of the ectal facet. The development of rugosities along the medial margin of the calcaneal tuberosity on NMB Eh 719 suggests that the calcaneal tendon might have had strong insertions along both the dorsal and medial surfaces of the tuber, as might be required if the calcaneus as a whole was typically held in an inverted position with respect to the tibia. The medial curvature, and moderately developed plantar projection, of the calcaneal tuber would also be expected to have increased the mechanical advantage of the superficial head of the flexor digitorum brevis muscle, which is important for species that engage in hind limb suspension because such an arrangement allows for strong digital flexion in diverse foot postures, notably those that might reduce the contractile potential of flexor fibularis (Sarmiento, 1983). A distinctly convex lateral border of the calcaneus is also seen (though to a greater degree) in lorisids, whereas dedicated leapers tend to have calcaneal tubers and distal segments whose long axes are roughly parallel. The mediolateral curvature of the calcaneal body in primates with more inverted feet might be functionally similar to dorsoplantar curvature of the calcaneal body in primates that have more everted feet; i.e., due to inversion, the medial surface of the tuber becomes functionally equivalent (in terms of the line of action of the triceps surae musculature) to the dorsal surface of the tuber in taxa with more everted feet.

Finally, we note that *Caenopithecus* has a particularly well-developed tubercle on the lateral surface of the astragalar neck for the astragalar-ectocuneiform ligament. This ligament tubercle is also well-developed in lorises, highly suspensory sloth lemurs such as *Babakotia* and *Palaeopropithecus*, the loris-like caenopithecine *Afradapis*, and the loris-like possible caenopithecine *Adapoides*, but is not clearly expressed to the same extent in adapines or grasp-leaping lemuriforms. We infer that the large tubercle reflects the existence of a particularly large astragalar-ectocuneiform ligament, and hypothesize that such an enlarged ligament might have been one of several ligamentous features that served (at least in part) to keep the astragalus closely anchored to the surrounding tarsals when the compressive load of the tibia on the astragalus was released—as would be expected to occur during hind limb suspension. Such a morphological adaptation would undoutedly be a more energy-efficient mechanism for maintaining articular integrity of the tarsals in habitually suspensory postures than to depend on muscles and their associated tendons. This interpretation calls into question whether *Notharctus* might have also utilized hind limb suspensory behaviors to a significant degree because it (but not *Cantius*) exhibits a tubercle that is developed to the same extent as that of *Caenopithecus*.

In summary, when all of the evidence presented here is taken into account, we consider it most likely that *Caenopithecus* had a tarsus that was habitually held in strong inversion, and that this species was capable of strong pedal grasping in a diversity of postures, as is typical of slow-moving primates that simultaneously use all four limbs to navigate arboreal settings in which branches are small relative to hand and foot size (such as terminal branches). We do not detect any compelling morphological evidence for habitual leaping or acrobatic grasp-leaping in *Caenopithecus*’ tarsals, but we do consider their morphology to be *consistent* with the use of hind limb suspension. Jenkins & McClearn (1984) pointed out that hind limb reversal of the sort that is required for hind limb suspension is accomplished in placental mammals via cruro-astragalar plantarflexion, subtalar inversion, and transverse tarsal supination, all of which we consider to have been possible in *Caenopithecus* and facilitated particularly well by its morphology. Inverted postures might be further indicated by a long and deep plantar exposure of the flexor fibularis groove, the development of the large tubercle for the astragalar-ectocuneiform ligament, and by the correlation of such extreme postures with slow climbing as suggested by the existence in *Caenopithecus* of relatively small tarsal facets (in this case estimated relative to tooth size—though admittedly this could also relate to a folivorous diet (and relatively large molars) as suggested above).

### *Lifestyle, phylogenetic position, and biogeographic origin of* Caenopithecus

If we are correct in attributing the Egerkingen tarsals to *Caenopithecus lemuroides*, then members of this species can now be reconstructed as having been folivorous slow climbers that were approximately 1.5–2.5 kg in body mass. We consider it unlikely that *C. lemuroides* individuals were adept leapers, and more probable that members of this species consistently maintained powerful grasps on branches as they moved through their arboreal habitats. We also consider it possible, based in part on the form-function correlation that we propose for the depth of the flexor fibularis groove on the plantar surface of the astragalus, that *C. lemuroides* might have regularly engaged in hind limb suspensory postures, though more evidence from the other bones of the hind limb is certainly needed to fully test that hypothesis. Regardless of whether the inference of hind limb suspension is correct, the reconstruction of *C. lemuroides* as a folivorous and slow climbing species suggests that the best extant primate analogues might be found among howler monkeys (*Alouatta*), although all known living and extinct alouattines are considerably larger than *C. lemuroides* (ranging in size from ∼4 to 11.5 kg; Smith & Jungers, 1997). The combination of folivory and slow climbing arguably also fits with what might be predicted for the adaptive profile of basal “stem” members of the lemuriform indrioid palaeopropithecid clade, prior to their acquisition of extreme specializations for quadrumanual under-branch suspension. The acquisition of such a lifestyle in distantly related lineages that presumably shared a common ancestor with distinctively strepsirrhine ankle morphology (Gebo, 2011; Boyer & Seiffert, 2013) may help to explain tarsal similarities that are shared by *C. lemuroides* and *Babakotia*.

Most of our phylogenetic analyses place *Caenopithecus* close to adapines to the exclusion of other non-caenopithecine adapiforms, but importantly the new tarsal evidence shows that *Caenopithecus* is not just a dentally aberrant adapid with an adapine-like postcranium; instead, *Caenopithecus* appears to be an adaptively unique member of Europe’s middle Eocene primate fauna. The biogeographic underpinnings of the adapiform diversity seen in the middle Eocene of Europe remain mysterious. Godinot (1998) argued that *Caenopithecus* and Adapinae were likely immigrant taxa that arrived in Europe independently during the middle Eocene, possibly from Asia, but simple parsimony reconstructions of continental biogeography onto the trees derived from our phylogenetic analyses all unambiguously imply that the last common ancestor of caenopithecines and adapines was already present in Europe. Nevertheless, a key taxon in Godinot’s scenario was middle Eocene *Adapoides* from the Shanghuang fissure fillings in China (Beard et al., 1994), which was not included in our phylogenetic analyses because undescribed material of that taxon announced in an abstract (Coster, Ni & Beard, 2012) indicates that teeth previously assigned to another adapiform in the Shanghuang fauna actually belong to *Adapoides*; we await explicit clarification on this matter before integrating *Adapoides* into phylogenetic analysis. Admittedly, the new observations (and expanded sample of *Adapoides* noted by Coster et al.) could have an important impact on our understanding of the biogeographic origins of adapines and caenopithecines.

The “core” caenopithecines (*Aframonius*, *Afradapis*, and *Caenopithecus*) present another biogeographic puzzle—depending on the assumptions and methods employed there were either (1) two unambiguously independent caenopithecine dispersals from Europe to account for the presence of *Aframonius* and *Afradapis* in Africa (i.e., on the two “allcompat” trees derived from the Bayesian analyses, and on that derived from parsimony analysis with all characters unordered), or (2) ambiguity, with two independent dispersals to Africa or back-migration of *Caenopithecus* to Europe being equally parsimonious (i.e., on the remaining trees). The biogeographic scenario is further complicated by the fact that the middle Eocene “caenopithecids” *Mescalerolemur* and *Mahgarita* from west Texas—which are unique records in North America, and arguably the most biogeographically anomalous adapiforms—do not form a clade in our Bayesian analyses, or in our parsimony analyses when characters are treated as unordered (these taxa do form a clade in our parsimony analyses, when some characters are treated as ordered). In our opinion, the separation of these genera (and the independent colonizations of North America required by such a result) is almost certainly erroneus and more likely reflects the *in situ* acquisition of caenopithecine-like convergences in *Mahgarita* (e.g., mandibular symphyseal fusion, enlarged upper molar hypocones, acquisition of an enlarged P^3^ protocone) from a *Mescalerolemur*-like form that more closely resembled anchomomyins or djebelemurids. The placement of *Mescalerolemur* as a close relative of crown strepsirrhines to the exclusion of non-anchomomyin adapiforms in our Bayesian analyses is similar to the placement retrieved by Kirk & Williams (2011) for a combined *Mescalerolemur-Mahgarita* clade when those authors employed a previous version of the matrix used here in their parsimony analyses (but notably with a reduced taxon set outside of Strepsirrhini). The divergent placements of *Mescalerolemur* and *Mahgarita* in some of our phylogenetic analyses clearly reveals the potential for parallel acquisition of the aforementioned caenopithecine-like features from more generalized small-bodied ancestors, and suggests that much more fossil evidence is needed from Afro-Arabia, Asia, Europe, and North America to tease apart the phylogenetic and biogeographic history of this group. Tarsals of *Mescalerolemur* or *Mahgarita* could provide particularly decisive evidence, given that *Adapoides*, *Afradapis*, and *Caenopithecus* are all known to have somewhat loris-like astragali (Gebo et al., 2001; Boyer, Seiffert & Simons, 2010) that differ markedly from those known for notharctids, djebelemurids, anchomomyins, and *Europolemur*.

Finally, we note that middle Eocene localities in Europe have now yielded primate and non-primate species whose adaptive profiles closely resemble those that might have been present among Madagascar’s lemuriforms in the later Oligocene and early Miocene, when non-daubentoniid clades were likely diversifying (Springer et al., 2012; Kistler et al., 2015). As discussed earlier, as slow moving folivores, caenopithecines might have resembled basal palaeopropithecids, while adapines and *Europolemur-*like forms were presumably more like basal lemurids in their lifestyles; anchomomyin stem strepsirrhines and microchoerine omomyiforms were cheirogaleid-like in being small-bodied insectivorous or omnivorous species capable of leaping (Dagosto & Schmid, 1996; Boyer et al., 2015b); and the non-primate apatemyids appear to have occupied a niche similar to that of extant *Daubentonia* (e.g., Koenigswald, 1990). A similar pattern might have existed slightly later in the middle Paleogene of Afro-Arabia, with the caenopithecine *Afradapis* occupying a basal palaeopropithecid-like niche, djebelemurids and basal lorisiforms occupying cheirogaleid-like niches, and plesiopithecids (based solely on craniodental evidence) possibly being somewhat *Daubentonia*-like (Godinot, 2006; Godinot, 2010). Afro-Arabia clearly differs from both Europe and (presumably) Madagascar, however, in having a diverse anthropoid fauna through the later Paleogene. The extent to which dispersal and/or endemic common ancestry shaped the similar adaptive composition of primate communities on these landmasses (which were largely isolated and thus biogeographically filtered during the later Paleogene) is a complex puzzle that will provide fascinating insight on primate evolutionary history as it is gradually pieced together by future work.

## Supplemental Information

10.7717/peerj.1036/supp-1 Appendix S1 Measurements on tarsal facets and lower second molars of extant and subfossil euarchontans that were used in regression analyses

10.7717/peerj.1036/supp-2 Appendix S2 Measurements on tarsal facets and lower second molars of Paleogene euarchontans that were used in regression analyses

10.7717/peerj.1036/supp-3 Appendix S3 Raw measurements and angular measurements, originally published by Boyer, Seiffert & Simons (2010) and augmented by Chester et al. (2015), taken on living and extinct euarchontans that were converted to shape variables and radians for principal components analysis Asterisks indicate which of the measurements were estimated on NMB En.270 due to abrasion or damage. More detailed explanations of how measurements are taken on digital models can be found in Boyer, Seiffert & Simons (2010).

10.7717/peerj.1036/supp-4 Appendix S4 Data from dental topography analysis of *Caenopithecus*, *Afradapis*, and 109 individuals from 21 extant “prosimian” genera Values are provided for the input variables M_2_ Relief Index (RFI), M_2_ Orientation Patch Count (OPC), and M_1_ area (length *x* width; note that the same M_1_ area (i.e., the mean M_1_ area of all M_1_*s* associated with M_2_*s*) is used for each M_2_ of *Afradapis* and *Caenopithecus*, because associated M_1_*s* were not available for each M_2_ analyzed); the dietary classification for extant taxa (“Actual group”); the group predicted by the discriminant function analysis (“Predicted group (highest prob.)”); probability of classification into the group predicted by the discriminant function analysis (“Probability”); next-best classification predicted by the discriminant function analysis (“Second highest group”); probability of the next-best classification predicted by the discriminant function analysis (“Probability (Second highest group)”); and the discriminant function scores for the first three discriminant axes (“DF1”, “DF2”, “DF3”). Results are based on a stepwise discriminant function analysis (using the Mahalanobis distance method) run in SPSS v. 22; because Box’s M test was significant, classifications were based on the group covariance matrices of the canonical discriminant functions, not the original variables. 93.6% of the original grouped cases were classified correctly. See Winchester et al. (2014) for a recent summary of the RFI and OPC methods and their efficacy.

10.7717/peerj.1036/supp-5 Figure S1 Plot of the first two discriminant functions based on RFI, OPC, and M_1_ area Individuals from each dietary category are enclosed by ellipses that include 95% confidence intervals (calculated in PAST, (Hammer, Harper & Ryan, 2001). Note that two specimens of *Afradapis* had the same RFI and OPC values and plot at the same points along DF1 and DF2.

10.7717/peerj.1036/supp-6 Dataset S1 Character-taxon matrix employed in the parsimony analysis with some multistate characters ordered and scaled Transitions between “fixed” states are equal to a single step (weights are provided in the PAUP block). Note that a constraint tree with the following topology must be enforced to obtain the results presented here: (*Tupaia glis*, (((((*Saimiri sciureus*, *Aotus trivirgatus*), *Alouatta seniculus*), (*Allenopithecus nigroviridis*, *Pan troglodytes*)), *Tarsius bancanus*), ((*Propithecus* spp., ((*Cheirogaleus major*, *Microcebus murinus*), *Lepilemur mustelinus*), (*Lemur catta*, *Varecia variegata*)), (((*Loris tardigradus*, *Nycticebus coucang*), (*Arctocebus calabarensis*, *Perodicticus potto*)), (*Galagoides demidoff*, (*Galago moholi*,*Otolemur crassicaudatus*)))))).

10.7717/peerj.1036/supp-7 Dataset S2 Constraint tree used in the parsimony analyses (also enforced as partial constraints in MrBayes)

10.7717/peerj.1036/supp-8 Dataset S3 3D coordinates for specimens analyzed in the automated geometric analysis of calcanei

10.7717/peerj.1036/supp-9 Dataset S4 Multidimensional Scaling coordinates derived from the automated geometric analysis of calcanei

## Institutional abbreviations

AMNH: American Museum of Natural History, New York, NY, USA
CM: Carnegie Museum of Natural History, Pittsburgh, Pennsylvania, USA
DPC: Duke Lemur Center Division of Fossil Primates, Durham, North Carolina, USA
ESC: Private collection of Mr. Dominique Vidalenc (Escamps locality)
GMH: Geiseltalmuseum, Halle, Germany
GU: H.N.B. Garhwal University, Srinagar, Uttarakhand, India
HTB: Hamann-Todd collection, Cleveland Museum of Natural History
IPS: Institut de Paleontologia de Sabadell (= Institut Català de Paleontologia Miquel Crusafont), Spain
ISEM: Institut des Sciences de l’Évolution de Montpellier, Montpellier, France (ISEM-ECA, Escamps locality, ISEM-BFI, La Bouffie locality)
IRSNB: Institut Royal des Sciences Naturelles del Belgique, Brussels, Belgium
IVPP: Insitute of Paleontology and Paleoanthropology, Chinese Academy of Sciences, Beijing, China
Ma-PhQ: Muséum d’Histoire Naturelle Victor Brun, Montauban, France
MCZ: Museum of Comparative Zoology, Harvard University, Cambridge, Massachusetts, USA
MNHN: Muséum National d’Histoire Naturelle, Paris, France
NMB: Naturhistorisches Museum Basel, Basel, Switzerland
NMNH: Smithsonian Institution National Museum of Natural History, Washington, D.C., USA
PMZ: Paleontological Museum of the University of Zürich, Zürich, Switzerland
ROS: Private collection of Mr. Dominique Vidalenc (Rosieres locality)
SBU: Department of Anatomical Sciences, Stony Brook University, Stony Brook, New York, USA
SDNHM: San Diego Natural History Museum, San Diego, California, USA
UCM: University of Colorado Museum of Natural History, Boulder, Colorado, USA
UCMP: University of California Museum of Paleontology, Berkeley, California, USA
UF: University of Florida, Florida Museum of Natural History, Gainesville, Florida, USA
UM: University of Michigan, Ann Arbor, Michigan, USA
USGS: U.S. Geological Survey, Denver, Colorado, USA
UNSM: University of Nebraska Science Museum, Lincoln, Nebraska, USA
USNM: United States National Museum, Smithsonian Institute, Washington D.C., USA
UWBM: Burke Museum, Seattle, Washington, USA
VPL/JU: Vertebrate Palaeontology Laboratory, University of Jammu
YPM: Yale Peabody Museum, Yale University, New Haven, Connecticut, USA

## Additional Information and Declarations

### Data Deposition

The following information was supplied regarding the deposition of related data: Digital models of the specimens described here are available on MorphoSource (www.morphosource.org). DOIs for the specimens are as follows: DOI 10.17602/M2/M5397 DOI 10.17602/M2/M5398 DOI 10.17602/M2/M5399 DOI 10.17602/M2/M5400 DOI 10.17602/M2/M5401 DOI 10.17602/M2/M5402 DOI 10.17602/M2/M5403 DOI 10.17602/M2/M5404.
