# Supplementary figures and images for: Primate tarsal bones from Egerkingen, Switzerland, attributable to the middle Eocene adapiform Caenopithecus lemuroides
Source: PeerJ. 2015 Jun 23;3:e1036. doi: 10.7717/peerj.1036 (PMC4485257; doi:10.7717/peerj.1036)

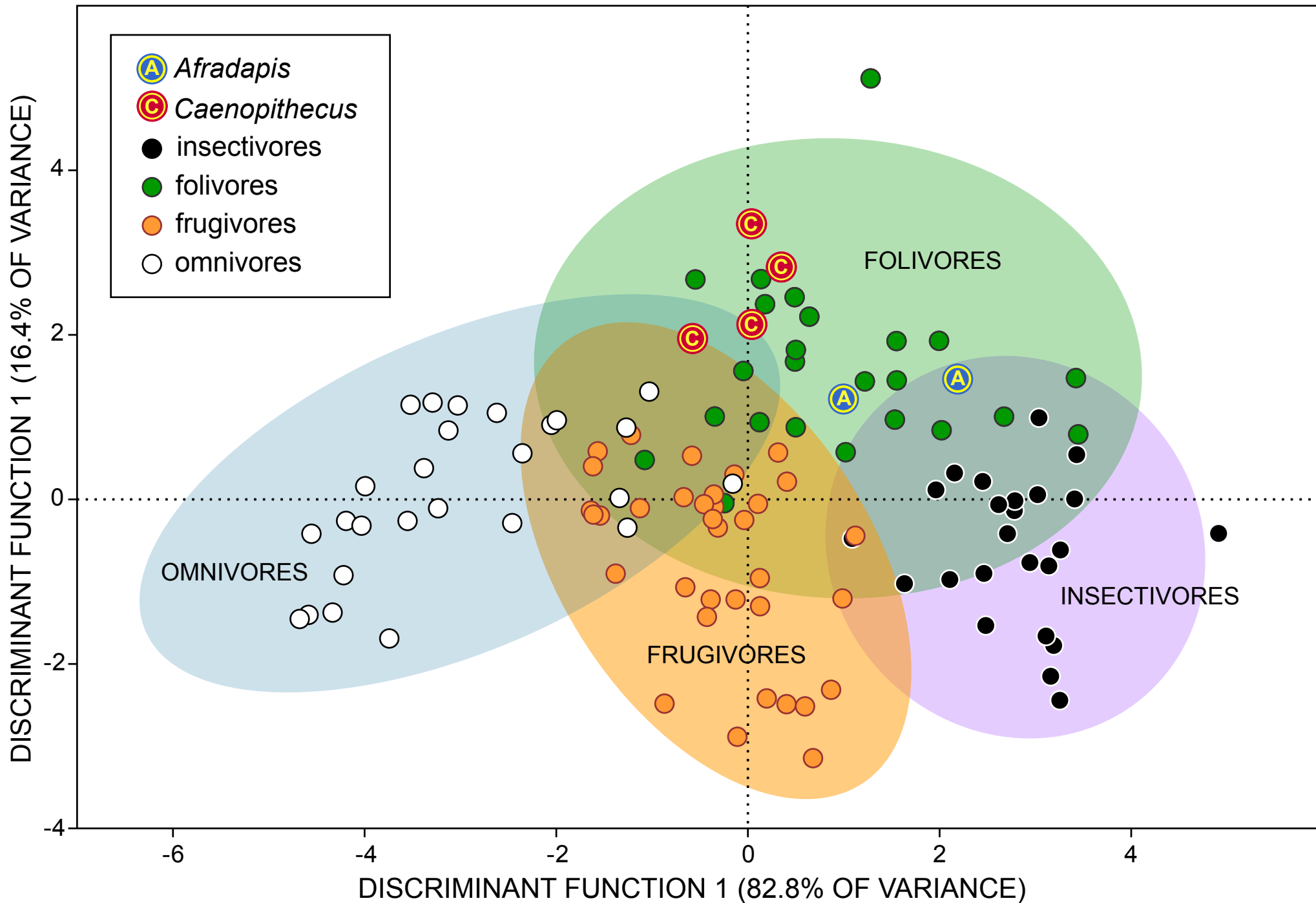

Supplement: Figure S1 — Individuals from each dietary category are enclosed by ellipses that include 95% confidence intervals (calculated in PAST, (Hammer, Harper & Ryan, 2001). Note that two specimens of Afradapis had the same RFI and OPC values and plot at the same points along DF1 and DF2. [file peerj-03-1036-s005.pdf]
